# Efficacy of a transition to oral antimicrobial therapy for gram-negative bacilli bloodstream infection: systematic review and meta-analysis

**DOI:** 10.1017/ash.2026.10742

**Published:** 2026-06-10

**Authors:** Hitoshi Honda, Toshiki Miwa, David Warren, Akane Takamatsu

**Affiliations:** 1 Fujita Health University School of Medicine, Japan; 2 University of Tokyo Hospital: Tokyo Daigaku Igakubu Fuzoku Byoin, Japan; 3 University of Nebraska Medical Center, USA; 4 Institute of Science Tokyo, Japan

## Abstract

**Background:**

While the disease and economic impact of gram-negative bacilli bloodstream infections (GNB-BSI) are high, treatment strategies, including transitioning from intravenous antimicrobial therapy (IVAT) to oral stepdown antimicrobial therapy (OSAT) are still in their nascent stage of development.

**Methods:**

A systematic review and meta-analysis evaluated the clinical effectiveness of OSAT compared with IVAT for GNB-BSI. Medline and Embase were searched from January 1980 to March 2025, along with the Cochrane Central Register of Controlled Trials, ClinicalTrials.gov, and reference lists of eligible studies. Randomized controlled trials and observational studies comparing OSAT with IVAT were included. Three investigators screened studies, extracted data, assessed study quality, and risk of bias, following PRISMA guidelines, ROBINS-E for observational studies and RoB 2 for randomized trials. Certainty of evidence was assessed using the GRADE framework.

**Results:**

Sixteen studies involving 4,858 patients who received OSAT and 4,961 patients who received IVAT were extracted for review. The pooled risk ratio for mortality and treatment failure in the primary analysis which included studies adjusting for confounders (n = 3) was 0.95 (95% confidence interval [CI]: 0.42, 2.14; *I* ^2^ = 8%) and 1.11 (95% CI: 0.22, 5.51; *I* ^2^ = 59%), respectively. The GRADE assessment for the primary analyses rated the evidence as very low to moderate certainty. Outcome definitions varied substantially across studies, potentially affecting pooled estimates.

**Conclusions:**

Although an imbalance in the baseline characteristics of patients with GNB-BSI across the studies warranted closer attention, OSAT may be as effective as IVAT for GNB-BSI without compromising clinical outcomes. Nevertheless, high-quality RCTs are needed before making definitive recommendations.

## Introduction

Gram-negative bacilli bloodstream infections (GNB-BSI) pose a significant threat in healthcare settings.^1^ GNB-BSI accounts for 40% to 60% of community-acquired and 30% to 40% of healthcare-associated BSI, with mortality ranging from 12% to 25%, and is particularly high among elderly populations and those with antimicrobial resistance, sepsis, and comorbidities.^2–5^ GNB-BSI is a clinical and public health concern given its burden and unfavorable prognosis.

Much attention has been given to strategies for managing GNB-BSI, particularly antimicrobial use. Recently, early transition to oral antimicrobial therapy (OSAT) for uncomplicated GNB-BSI originating from urinary tract infections (UTI) has emerged as a promising approach, offering potential benefits such as reducing healthcare costs, earlier hospital discharge, and improving patient tolerability without compromising outcomes.^6–10^

However, the effectiveness of OSAT must be evaluated through clinical outcomes such as treatment success, treatment failure, and length of hospital stay (LOHS) to ensure it is comparable to intravenous antimicrobial therapy (IVAT). Existing systematic reviews also have notable gaps: one did not include a recent randomized trial and high-quality observational studies,^11^ while another examined BSI broadly, rather than focusing on GNB-BSI.^12^

To address these limitations, we conducted a systematic review and meta-analysis comparing OSAT with IVAT for GNB-BSI. The primary meta-analysis included studies that adjusted for confounding variables affecting these outcomes, while a sensitivity (expanded) analysis incorporated all relevant studies, regardless of whether the aforementioned adjustments had been made. This review aimed to determine whether OSAT is an appropriate and effective alternative to IVAT for GNB-BSI.

## Methods

The present systematic review and meta-analysis was conducted in accordance with the Cochrane Handbook for Systematic Reviews of Interventions. Documentation of reports followed the Preferred Reporting Items for Systematic Reviews and Meta-analyses (PRISMA) statement.^13^ The protocol of the present systematic review was prospectively registered in PROSPERO (CRD420251005994) and updated to the latest version.^14^

### Data sources and searches

Studies published on Medline (Ovid) and Embase (Ovid) between January 1980 and March 2025 were reviewed for information relevant to OSAT for GNB-BSI. The Cochrane Central Register of Controlled Trials and ClinicalTrials.gov. were also searched to identify potentially eligible trials. In addition, the reference lists of the relevant systematic reviews, narrative reviews, and articles subsequently included in the present systematic review and meta-analysis were scrutinized. The search terms incorporated multiple, controlled vocabularies (eg, Medical Subject Headings), including “oral stepdown therapy,” “bacteremia,” and “gram-negative bacilli” (Supplementary Table 1). A search of the reference list, Cochrane Central Register of Controlled Trials, and ClinicalTrials.gov was conducted by the primary reviewer (H.H.). Potentially eligible articles were initially uploaded to EndNote 21, and the data were then converted for uploading to the Rayyan Systematic Review software.^15,16^

### Study selection

Eligible studies for this systematic review were comparative investigations evaluating the clinical efficacy of IVAT versus OSAT for treating adult patients with GNB-BSI, including those caused by Enterobacterales and lactose-nonfermenting organisms. Using the PI(E)CO framework, (P) the population consisted of adults aged ≥15 years; (I or E) the intervention or exposure was OSAT; (C) the comparator was continuous IVAT; and (O) the outcomes were mortality and treatment failure. Only English-language studies were included. Duplicates were removed by the primary reviewer (H.H.) with the assistance of a duplication and systematic resolver tool included in the Rayyan software. In the screening process, three reviewers (H.H., T.M., and A.T.) independently vetted titles and abstracts of all the potentially eligible items. Abstracts not conforming to the eligibility criteria were excluded. After the initial screening, all the selected research articles were retrieved for a full-text review, which three reviewers (H.H., T.M., and A.T.) independently vetted to determine which articles should be included in the systematic review. The reviewers resolved any differences through discussion and simultaneous, full-text reviews in an online meeting.

### Data extraction and quality assessment

The primary reviewer (H.H.) extracted all data relevant to the systematic review, and three reviewers (H.H., T.M., A.T.) independently extracted all the outcome data of each study. Outcome data included event counts and sample sizes for dichotomous variables. The three reviewers independently assessed observational studies using the Risk Of Bias In Non-randomized Studies of Exposures (ROBINS-E: 7 domains) and randomized trials using the Risk Of Bias 2 (RoB 2: 5 domains).^17,18^ ROBINS-E ratings were “low,” “some concerns,” “high,” or “very high,” while RoB 2 used “low,” “some concerns,” or “high.” Domain ratings were aggregated to determine overall risk of bias, and disagreements were resolved through discussion. Three studies with a conference abstract were not assessed for risk of bias because of insufficient information about these studies.

The primary reviewer (H.H.) rated the overall certainty of the evidence of the key outcomes in the primary meta-analysis using the Grading of Recommendations Assessment, Development and Evaluation (GRADE) framework.^19^ Evidence quality was graded across five domains: study design, risk of bias, inconsistency, indirectness, and imprecision as defined by the guidance in the Cochrane Handbook for Systematic Reviews of Interventions.^20^ Since the GRADE framework assessment could not be applied for the meta-analysis compiling different types of studies (ie, both RCTs and observational studies), it was separately applied to meta-analyses by including only RCTs and observational studies for each outcome. GRADEpro GDT was used to create the GRADE evidence profile.^21^

### Data synthesis and analysis

A meta-analysis incorporating dichotomous and continuous outcomes was conducted as sufficient outcome data became available. Mortality data were synthesized as reported despite the varying timeframes (eg, in-hospital, 30-, 60-, or 90-day mortality). Although the definition of treatment failure also varied (eg, recurrence or antimicrobial resumption), it was adapted to the specific needs of this study. Table 1 provides the definition of mortality and treatment failure found in the selected studies.

**Table 1.** Definition of outcomes of interest in each study eligible for the systematic review and meta-analysis

First author, year	Definition of mortality	Definition of treatment failure
Rieger KL, 2017	In-hospital all-cause mortality	Escalation to IV from PO antimicrobials, change in antimicrobials due to worsening clinical status or readmission for the same infection/pathogen (either UTI and/or bacteremia) within 30 days of discharge.
Meije Y, 2019	30-day all-cause mortality	Bacteremia persistence, recurrence or relapse.
Savage H, 2021	Unavailable	Treatment failure was defined as an escalation from PO to IV antimicrobials, worsening clinical status or readmission for the same infection within 30 days of discharge.
Tamma PD, 2019	30-day all-cause mortality	Not stated. However, data on 30-day bacteremia recurrence were available and were adopted in the quantitative synthesis of treatment failure.
Williams K, 2019	Unavailable	Not stated. Since the treatment cure rate was available, the number of treatment failures was subtracted from the total number of cases to determine the treatment failure rate, which was then used to perform the qualitative synthesis of treatment failure.
Mulvey N, 2021	Unavailable	Treatment failure was not defined although the incidence of treatment failure was available.
Pradubkham T, 2021	In-hospital all-cause mortality	Composite outcome including BSI relapse and return from oral to IV medication (in-hospital mortality was excluded from the quantitative synthesis of treatment failure).
Tossey JC, 2021	In-hospital all-cause mortality	Infection recurrence or infection-related readmission.
Noguchi T, 2022	60-day all-cause mortality	Recurrence of infection within 60 days of BSI.
Avila-Nunez M, 2023	Unavailable	Not stated. However, data on infection recurrence were available and were adopted in the quantitative synthesis of treatment failure.
Nguyen N, 2023	30-day all-cause mortality	Not stated. However, data on 30-day bacteremia recurrence were available and were adopted in the quantitative synthesis of treatment failure.
Engers DW, 2024	Unavailable	Not stated. However, the 30-day readmission rate was available and was adopted in the quantitative synthesis of treatment failure.
Nussbaum EZ, 2024	30-day all-cause mortality	Not stated. However, data on 30-day bacteremia recurrence (the primary outcome measure) were adopted in the quantitative synthesis of treatment failure.
Omrani AS, 2024	90-day all-cause mortality	Composite outcome of (a) death of any cause, (b) need for additional antimicrobial therapy with one or more microbiologically active agents, (c) microbiological relapse, and (d) infection-related re-admission all within 90 days of starting microbiologically active antimicrobial therapy (mortality data were removed for the quantitative synthesis of treatment failure).
Tingsgård S, 2024	90-day all-cause mortality	Not stated. Data on treatment failure unavailable
Veillette JJ, 2024	90-day all-cause mortality	Not stated. However, data on 30-day bacteremia recurrence (the primary outcome measure) were adopted in the quantitative synthesis of treatment failure.

IV, intravenous; PO, per os; UTI, urinary tract infection; BSI, bloodstream infection.

Data synthesis was conducted using Cochrane’s Review Manager (RevMan).^22^ The risk ratio (RR) with 95% confidence intervals (CIs) were calculated for dichotomous outcomes using the Mantel-Haenszel random-effects model. All models employed the Hartung-Knapp-Sidik-Jonkman approach, which applies to the DerSimonian-Laird estimator to obtain less biased estimated effects and 95% CI.^23^ *P* < .05 was considered to indicate statistical significance. To minimize the bias from confounding, the primary meta-analysis included only a RCT and confounders-adjusted observational studies. Due to the small number of studies (n = 3) in the primary analysis, publication bias was not assessed. Heterogeneity was evaluated using forest plots and the *I* ^2^ statistic interpreted as 0%–40% (unimportant), 30%–60% (moderate), 50%–90% (substantial), and 75%–100% (considerable).^24^

### Sensitivity (expanded) analyses and subgroup analyses

Sensitivity and subgroup analyses were conducted to assess model robustness for key outcomes. The sensitivity (expended) analysis also incorporated studies whose findings had not been adjusted for confounders. Publication bias was evaluated using Funnel plot with visual inspection. Additional, discrete, subgroup analyses examined 1) studies involving immunocompromised patients, 2) studies only including drug-resistant pathogens (Amp C or extended-spectrum beta-lactamase-producing Enterobacterales), 3) studies with oral transition within 5 days of IVAT, 4) studies assessing 30-day mortality, and 5) studies focusing solely on GNB-BSI originating from UTIs.

## Results

The initial database search identified 6,068 studies that were potentially eligible for inclusion in the present systematic review and meta-analysis. After independent title and abstract screening, 83 remained for full-text review. Before the review was conducted, a review article, a protocol study, and a pediatric study (n = 3) were excluded. Of the remaining 80 articles, 11 met the inclusion criteria. Also identified as potentially eligible for the systematic review and meta-analysis were 17 additional studies found in reference lists and trial registries, of which five were included, yielding 16 total studies. Supplementary Table 2 summarizes the reasons for exclusion. Supplementary Figure 1 shows the PRISMA flow diagram, which identified 16 studies. Supplementary Table 3 shows the PRISMA statement.

### Characteristics of the included studies

A total of 16 studies (9,819 patients) subsequently met the inclusion criteria for the present systematic review and meta-analysis, and those were published from 2017 to 2024. Of these, one (6%) was a multi-center, open-label RCT,^7^ six (38%) were multi-center, retrospective cohort studies,^6,25–29^ and the remaining nine (56%) were single-center, retrospective cohort studies.^30–38^ Seven studies (44%) included a mixed population (with immunocompromised patients [ie, hematologic malignancies, solid organ and bone marrow transplant, and human immunodeficiency virus infection] accounting for >10% of the overall study population),^6,25,26,29,30,34,38^ four studies (25%) included only non-immunocompromised patients,^7,27,36,37^ and two (13%) included only immunocompromised patients.^28,31^ The primary source of BSI varied to include UTI, intra-abdominal infection, and catheter-related bloodstream infection (CRBSI). Of these, UTI was the most common, and four studies (25%) solely included patients with bacteremic UTI.^26,27,33,36^ *Escherichia coli* was the most common causative pathogen of GNB-BSI. Eight (50%) studies revealed that patients with IVAT had more severe GNB-BSI (eg, a higher Pitt bacteremia score or ICU admission) and a higher proportion of underlying illnesses (ie, a higher Charlson comorbidity index) than those with OSAT. Differences in patients’ characteristics between IVAT and OSAT are shown in Supplementary Table 4.

Only six studies (38%) had a precisely defined cut-off ranging from three to ten days for the transition to OSAT.^6,7,25–27,29^ Twelve studies documented the actual duration to the transition from IVAT to OSAT, ranging from 2.5 days (IQR: 0, 6) to 7.0 days (IQR: 5.8, 9.4).^6,7,25–29,34–36,38^ Fluoroquinolones were the most common oral stepdown antimicrobial, followed by trimethoprim-sulfamethoxazole (Supplementary Table 4). Eleven studies (69%) reported mortality outcomes: four on 30-day,^6,28,34,35^ three on 90-day,^7,25,26^ three on in-hospital,^30,36,38^ and one on 60-day mortality.^27^ Fifteen studies (94%) provided treatment failure rates, which varied across studies. All eleven studies reporting hospitalization duration showed shorter LOHS in the OSAT group.^6,7,26–30,35–38^ Supplementary Table 5 summarizes crude mortality, treatment failure and LOHS. Funnel plot inspection revealed no significant publication bias (Supplementary Figure 2 [2A, 2B]).

Among 16 studies, three (19%) were included in the primary analysis: (1) a multicenter, open-label RCT in which *E. coli* comprised 65% of GNB-BSI and UTIs were the source in 60% of cases; (2) a multicenter retrospective cohort study in which *E. coli* comprised 43% of GNB-BSI and 40% originated from UTIs, using propensity score matching to adjust baseline characteristics; and (3) a single-center retrospective cohort study in which *E. coli* comprised 60% of GNB-BSI and 60% originated from UTIs, also using propensity score matching.

### Risk of bias assessment

Of the sixteen studies, three studies^6,7,25^ had a low risk of bias, four studies^27,30,34,38^ had “some concerns” for a risk of bias, and the remaining six studies^26,28,29,35–37^ had a “critical” risk of bias. The last two groups did not control confounders and were biased in their choice of the findings they reported. Moreover, a considerable proportion of single-center, retrospective studies contained some risk of bias. Supplementary Figure 3 shows the domain level of the risk of bias.

### Main analysis

#### Mortality

**Figure 1.**
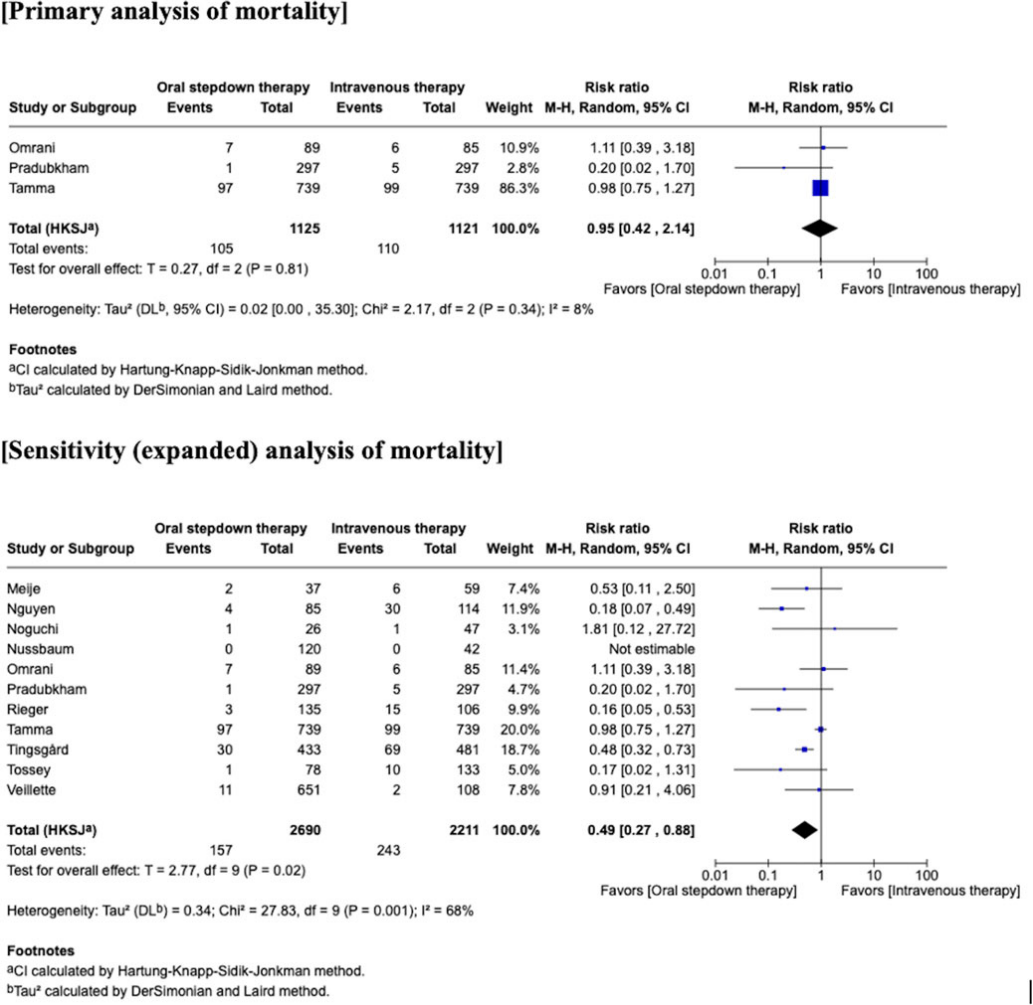
Forest plots of the random-effect meta-analysis for the mortality rate in patients with a bloodstream infection due to gram-negative bacilli which was treated with either oral step-down antimicrobial therapy or intravenous antimicrobial therapy.

The primary meta-analysis for all-cause mortality included only cohorts that had been adjusted for confounders (n = 3).^6,7,30^ The pooled estimate for OSAT versus IVAT had a RR of .95 ([95% CI: .42, 2.14]; *I* ^2^ = 8%; moderate certainty of evidence from RCT and low certainty of evidence from observational studies), indicating that OSAT was not associated with increased mortality. In the sensitivity analysis, which included studies irrespective of adjustment for confounders, (n = 11), the pooled estimate of mortality favored OSAT (RR: 0.49 [95% CI: 0.27, 0.88]; *I* ^2^ = 68%; moderate certainty of evidence from RCT and very low certainty of evidence from observational studies).^6,7,25–28,30,34–36,38^ Figure 1 shows the forest plots for both analyses and Table 2 shows the GRADE assessment for the meta-analysis of mortality.

**Table 2.** Grading of Recommendations Assessment, Development and Evaluation (GRADE) of oral stepdown therapy versus intravenous antimicrobial therapy for bloodstream infection due to gram-negative bacilli

The GRADE assessment for primary meta-analysis												Importance
Certainty assessment							№ of patients		Effect		Certainty	
№ of studies	Study design	Risk of bias	Inconsistency	Indirectness	Imprecision	Other considerations	Oral stepdown therapy	Intravenous therapy	Relative (95% CI)	Absolute (95% CI)		
**Mortality (only one randomized trial included in the primary analysis)**												
1	Randomized trials	Serious^a^	Not serious	Not serious	Not serious	None	7/89 (7.9%)	6/85 (7.1%)	**RR 1.11** (0.39 to 3.18)	**8 more per 1,000** (from 43 fewer to 154 more)	⨁⨁⨁◯ Moderate^a^	CRITICAL
**Mortality (only observational studies included in the primary analysis)**												
2	Non-randomized studies	Not serious	Not serious	Not serious	Not serious	None	98/1036 (9.5%)	104/1036 (10.0%)	**RR 0.64** (0.00 to 4,932.71)	**36 fewer per 1,000** (from -- to 1,000 more)	⨁⨁◯◯ Low	CRITICAL
**Treatment failure (only one randomized trial included in the primary analysis)**												
1	Randomized trials	Serious^a^	Not serious	Not serious	Not serious	None	15/89 (16.9%)	18/85 (21.2%)	**RR 0.80** (0.43 to 1.48)	**42 fewer per 1,000** (from 121 fewer to 102 more)	⨁⨁⨁◯ Moderate^a^	CRITICAL
**Treatment failure (only observational studies included in the primary analysis)**												
2	Non-randomized studies	Not serious	Serious^b^	Not serious	Serious^c^	None	24/1036 (2.3%)	15/1036 (1.4%)	**RR 1.36** (0.00 to 2,514.03)	**5 more per 1,000** (from -- to 1,000 more)	⨁◯◯◯ Very low^b^ ^,^ ^c^	CRITICAL
The GRADE assessment for sensitivity (expanded) meta-analysis												
**Mortality (only observational studies included in the sensitivity analysis)**												
10	Non-randomized studies	Very serious^d^	Serious^e^	Not serious	Not serious	All plausible residual confounding would suggest spurious effect, while no effect was observed	150/2601 (5.8%)	237/2126 (11.1%)	**RR 0.44** (0.23 to 0.81)	**62 fewer per 1,000** (from 86 fewer to 21 fewer)	⨁◯◯◯ Very low^d, e^	CRITICAL
**Treatment failure (only observational studies included in the sensitivity analysis)**												
14	Non-randomized studies	Very serious^d^	Serious^e^	Not serious	Not serious	All plausible residual confounding would suggest spurious effect, while no effect was observed	480/4336 (11.1%)	670/4395 (15.2%)	**RR 0.72** (0.51 to 1.01)	**43 fewer per 1,000** (from 75 fewer to 2 more)	⨁◯◯◯ Very low^d^ ^,^ ^e^	CRITICAL

CI, confidence interval; MD, mean difference, RR, risk ratio.

a The risk of bias was downgraded because an open-label trial was included.

b Precision was downgraded because few events occurred in either group.

c, e Inconsistency was downgraded because the results of the studies varied.

d Risk of bias was downgraded due to a lack of adjustment for potential confounders.

#### Treatment failure

**Figure 2.**
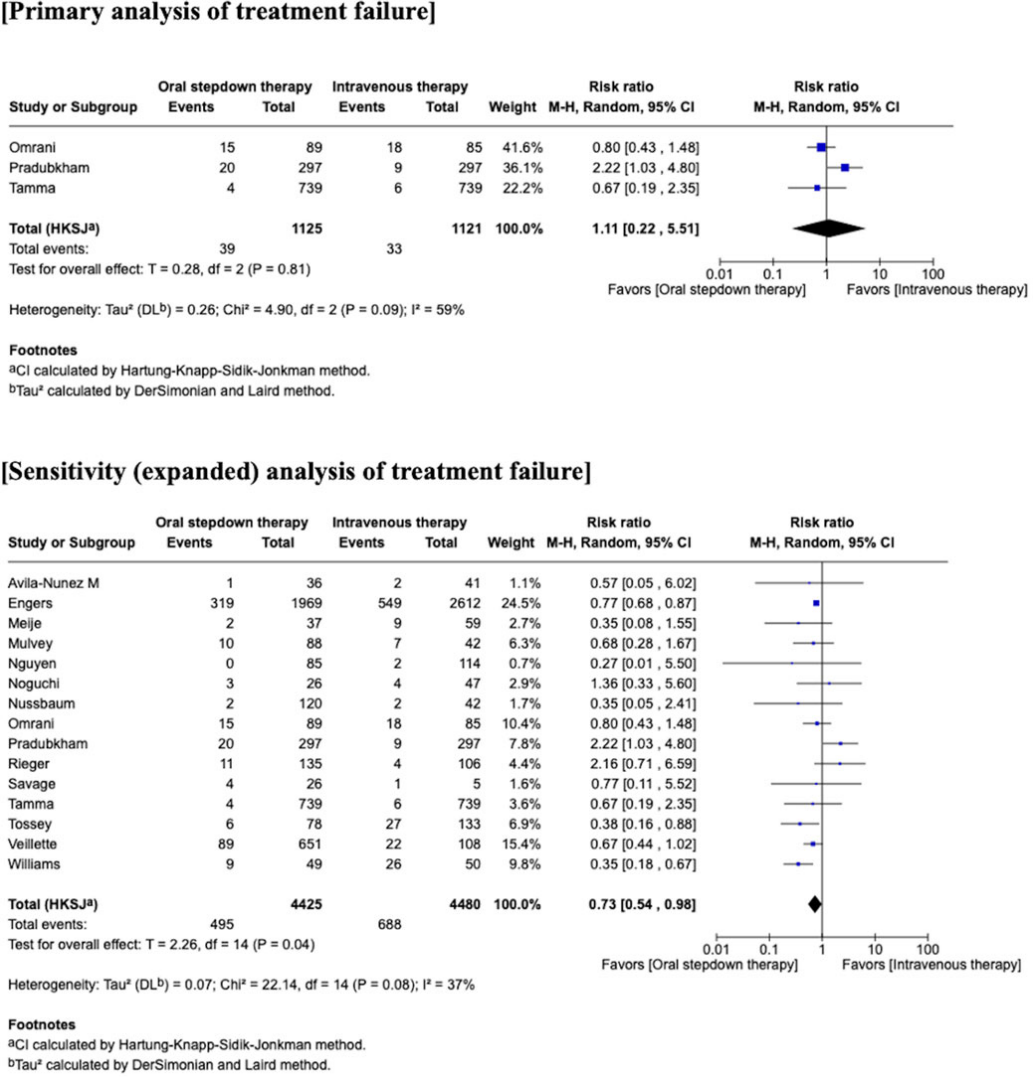
Forest plots of the random effects meta-analysis for the treatment failure rate in patients with bloodstream infection due to gram-negative bacilli which was treated with either oral step-down antimicrobial therapy or intravenous antimicrobial therapy.

Treatment failure was evaluated in fifteen studies (94%).^6,7,26–38^ The pooled RR for OSAT versus IVAT in the primary analysis (n = 3) was 1.11 (95% CI: 0.22–5.51; *I* ^2^ = 59%; moderate certainty evidence from RCT and very low certainty evidence from observational studies).^6,7,30^ In the sensitivity analysis, which included studies irrespective of adjustment for confounders (n = 15), the pooled RR favored OSAT (RR: 0.73 [95% CI: 0.54–0.98]; *I* ^2^ = 37%; moderate certainty evidence from RCT and very low certainty evidence from observational studies).^6,7,26–38^ Figure 2 shows forest plots for the primary and sensitivity analyses. Table 2 shows the GRADE assessment for the meta-analysis of the treatment failure rate.

#### Subgroup analysis

We performed five subgroup analyses. First, studies in which immunocompromised patients comprised more than 10% of the population showed a pooled mortality RR for the OSAT was 0.60 (95% CI: 0.34–1.11; *I* ^2^ = 59%).^6,25,26,28–31,34,38^ Second, studies limited to drug-resistant pathogens revealed a pooled mortality RR of 0.72 (95% CI, 0.00–564.30; *I* ^2^ = 0%).^27,34^ Third, subgroup analysis focusing on studies with transition to OSAT within 5 days (median or mean) demonstrated a pooled mortality RR of 0.60 (95% CI, 0.25–1.43; *I* ^2^ = 75%).^6,7,25,28,34,36^ The fourth subgroup analysis for studies with 30-day mortality revealed a pooled mortality RR of 0.95 (95% CI, 0.61–1.49; *I* ^2^ = 0%).^6,28,34,35^ The fifth subgroup analysis was stratified by studies focused solely on bacteremic UTI, revealed a pooled mortality RR of 0.49 (95% CI, 0.02–10.91; *I* ^2^ = 57%) for the OSAT group.^26,27,36^ Supplementary Table 6 and Supplementary Figures 4, 5, 6, 7, and 8 summarize the remaining pooled results and forest plots in the subgroup analyses. Supplementary Table 7 shows the GRADE assessment.

## Discussion

The present systematic review compared the efficacy of OSAT with that of IV antimicrobial therapy in treating GNB-BSI. Due to significant variations in the outcome measurements, study methodology, and clinical characteristics of the study populations, previous studies were able only to ascertain whether OSAT could be as effective as IVAT without compromising patient outcomes. Our systematic review comprehensively assessed 16 studies, including a recently published RCT and various observational studies focusing solely on GNB-BSI. Moreover, multiple sensitivity and subgroup analyses enhanced result granularity, providing a clearer understanding of OSAT effectiveness relative to IVAT.

Our primary analysis found that OSAT was not associated with increased mortality. Sensitivity and subgroup analyses, one incorporating all eligible studies and another focusing on 30-day mortality also did not demonstrate an increase in mortality in the OSAT cohort, which may corroborate the clinical efficacy of this treatment for GNB-BSI. Differences in the mortality effect size in the results of the primary and sensitivity analyses likely stemmed from unadjusted baseline characteristics and severity of BSI. Nevertheless, the true effect size for OSAT in treating GNB-BSI comparing with IVAT in the primary analysis remains uncertain. This uncertainty reflected the inclusion of only three studies (one RCT and two observational studies), its effect size with wide confidence interval, low to moderate overall certainty of the evidence, despite the low heterogeneity. These findings represent inconclusive evidence rather than the definitive proof of a compatible efficacy of OSAT to IVAT.

While concerns remained about vulnerable populations having poorer outcomes, our subgroup analysis of involving immunocompromised patients found no statistical difference in the mortality rate between the OSAT and IVAT cohorts. These findings suggested that OSAT may be safe also for high-risk groups. However, caution is warranted because studies with a low risk of bias required hemodynamic stability and adequate source control before effecting the transition to oral therapy, indicating that these are important considerations in clinical decision-making.^6,7^

The pooled risk of treatment failure in the primary analysis did not statistically differ between the OSAT and IVAT. Conversely, the sensitivity analysis found that OSAT conferred some protection against treatment failure. However, interpretation of OSAT treatment failure risk also requires caution, as primary studies may contain residual confounding, including inadequate empiric therapy, while sensitivity analyses may overestimate effects because of a lack of adjustment for key confounders that could influence outcome estimates in comparative analyses.

With respect to the LOHS, although all eleven studies reporting hospitalization duration showed shorter LOHS in the OSAT, these findings warrant cautious interpretation because the link between the duration of hospitalization and clinical outcomes is complex and subject to multiple confounding factors. Nevertheless, since a short duration may lower the risk of healthcare-associated complications,^39–41^ OSAT may be considered to improve outcomes and reduce healthcare costs and complications, given the large number of patients with a GNB-BSI in the healthcare setting.

The strengths of this systematic review include the use of a recent randomized trial and high-quality observational studies, standardized methodology with the use of objective risk-of-bias tools, GRADE-based certainty of evidence assessments, and multiple sensitivity and subgroup analyses to enhance robustness and precision. Our findings align with a recent guideline endorsing early OSAT for bacteremic UTI patients who are afebrile, hemodynamically stable, and have achieved source control.^42^ Its limitations include considerable variation in the definition of outcomes, particularly the mortality time frame and treatment failure, which may have biased the pooled estimates. Although OSAT efficacy should be stratified by infection source, causative organisms, and resistance patterns to optimize treatment across diverse clinical scenarios, the substantial heterogeneity of the studies prevented this detailed analysis. The relationship between changes in antimicrobial resistance rates to oral antimicrobial agents, especially fluoroquinolones on clinical outcomes remains unevaluated. The subgroup results focusing on GNB-BSI from a urinary source or involving multidrug-resistant pathogens may still offer a beneficial understanding of OSAT’s effectiveness in these selected populations. The optimal strategy for administering OSAT, including its choice of antimicrobial agent, remains unclear due to the small number of RCTs and high-quality observational studies. Moreover, prescribing patterns, including duration of OSAT, antimicrobial dosage, and selections, were highly heterogenous across the included studies. Determining the ideal timing of transitioning to oral therapy on the basis of current evidence was challenging. In fact, one multi-center, observational study found considerable variation in the timing of OSAT.^29^ Finally, the findings do not apply to GNB-BSI caused by highly drug-resistant organisms, such as carbapenem-resistant Enterobacterales. Of note, two ongoing randomized controlled trials (RCTs) are investigating the efficacy of OSAT, with the aim of addressing unresolved clinical questions.^43,44^

In conclusion, Although the observed imbalances in baseline characteristics among patients with GNB-BSI across the included studies required more careful consideration, OSAT may be as effective against GNB-BSI as IVAT without increasing the mortality rate. As antimicrobial stewardship programs continue to evolve, the strategic implementation of OSAT becomes more pivotal in optimizing patient outcome. OSAT may also be administered to immunocompromised patients if they are clinically stable with the following empiric IVAT. Patients with bacteremic UTI, who comprise a significant proportion of patients with GNB-BSI, may be ideal candidates for OSAT. Nevertheless, given the limited availability of the strong evidence in the present meta-analysis, high-quality RCTs are needed to confirm the efficacy of OSAT.

## Supporting information

10.1017/ash.2026.10742.sm001 Honda et al. supplementary material Honda et al. supplementary material

## Supplementary material

The supplementary material for this article can be found at https://doi.org/10.1017/ash.2026.10742.

## Reproducible research statement

The study protocol may be viewed at https://www.crd.york.ac.uk/PROSPERO/view/CRD420251005994. The statistical data set are available from Hitoshi Honda, M.D. (hitoshi.honda@fujita-hu.ac.jp).
