# Supplementary material for: Efficacy of a transition to oral antimicrobial therapy for gram-negative bacilli bloodstream infection: systematic review and meta-analysis
Source: Antimicrob Steward Healthc Epidemiol. 2026 Jun 10;6(1):e172. doi: 10.1017/ash.2026.10742 (PMC13273156; doi:10.1017/ash.2026.10742)

**Supplementary material**

Efficacy of a transition to oral antimicrobial therapy for gram-negative bacilli bloodstream infection: systematic review and meta-analysis

**Table of Contents**

**Supplementary Table 1.** Search terms for MEDLINE (OVID) and EMBASE (OVID)..……….4

**Supplementary Table 2.** Articles excluded from the systematic review

in order of publication year………………………………………………………………………..6

**Supplementary Table 3.** The Preferred Reporting Items for Systematic reviews

and Meta-Analyses (PRIMSA) statement checklist………………………..……….……………11

**Supplementary Table 4.** Characteristics of studies included in the systematic review

and meta-analysis………………………………………………………………………………...14

**Supplementary Table 5.** Crude mortality rate, treatment failure, and length of hospital stay in the studies included in the systematic review and meta-analysis…………………….………….19

**Supplementary Table 6.** Summary of the pooled results of the subgroup analyses…..……….20

**Supplementary Table 7.** Grading of Recommendations Assessment, Development and Evaluation (GRADE) for subgroup analyses…………………………………………………….21

**Supplementary Figure 1.** The Preferred Reporting Items for Systematic reviews and Meta-Analyses (PRIMSA) flow diagram for the present systematic review, which included searches

of databases, registers, and other sources.…..…………………………………………………...23

**Supplementary Figure 2.** Funnel plots of the sensitivity analysis………..…………………….24

　　 2A. A funnel plot of the mortality rate in 11 studies with 10 effect estimates

in the sensitivity analysis………………………………………………………………24

2B. A funnel plot of the treatment failure rate in 15 studies with 15 effect

estimates in the sensitivity analysis…………………………………………………….25

**Supplementary Figure 3.** Risk of bias in the studies included in the meta-analysis…………...26

**Supplementary Figure 4.** Forest plots of the random-effects meta-analysis of the outcomes in the subgroup analysis (immunocompromised patients)...………………………………………..27

**Supplementary Figure 5.** Forest plots of the random-effects meta-analysis of the outcomes in the subgroup analysis (studies only including multidrug-resistant organisms).…………………28

**Supplementary Figure 6.** Forest plots of the random-effects meta-analysis of the outcomes in the subgroup analysis (studies with oral transition within 5 days)………………………………29

**Supplementary Figure 7.** Forest plots of the random-effects meta-analysis of the outcomes in the subgroup analysis (focusing on studies with 30-day mortality)…..…………………………30

**Supplementary Figure 8.** Forest plots of the random-effects meta-analysis of the outcomes in the subgroup analysis (bacteremic urinary tract infection)………………………………………31

**Supplementary Table 1.** Search terms for MEDLINE (OVID) and EMBASE (OVID)

| **Database** | **Number** | **Search strategy** | **Result, N,**  (31-Mar-2025) |
| --- | --- | --- | --- |
| **MEDLINE (OVID)** | 1 | exp Bacteremia/ or exp Sepsis/ or (bloodstream infection* or bacteremia or septicemia).ti,ab,kf | 168473 |
|  | 2 | exp Gram-Negative Bacterial Infections/ or exp Gram negative bacterial infection/ or exp Enterobacteriaceae/ or exp Enterobacterales/ or exp Pseudomonas Infections/ or exp Acinetobacter Infections/ or (Gram-negative bacill* or Gram-negative rod* or Enterobacteriaceae or Enterobacterale* or Escherichia coli or Klebsiella or Pseudomonas or Acinetobacter or Proteus or Enterobacter or Serratia).ti,ab,kf. | 851228 |
|  | 3 | 1 and 2 | 31684 |
|  | 4 | exp Anti-Bacterial Agents/ or (antimicrobial* or antibiotic* or anti-infective*).ti,ab,kf. | 990855 |
|  | 5 | (oral stepdown or switch therap* or oral antibiotic* or step-down therap*).ti,ab,kf. | 6522 |
|  | 6 | 4 or 5. | 991125 |
|  | 7 | randomized controlled [trial.pt](http://trial.pt/). or randomized.ti,ab. or placebo.ti,ab. or clinical [trial.pt](http://trial.pt/). or (random* adj5 (assign* or allocat*)).ti,ab. | 1312359 |
|  | 8 | cohort [studies.pt](http://studies.pt/). or cohort*.ti,ab. or (follow-up or longitudinal or prospective or retrospective).ti,ab. or (incidence or risk or predict* or prognos*).ti,ab. | 6610749 |
|  | 9 | case-control [studies.pt](http://studies.pt/). or (case-control or case referent).ti,ab. or (retrospective adj3 (study or analysis)).ti,ab. or (odds ratio or matched or control group).ti,ab. | 1622781 |
|  | 10 | 7 or 8 or 9 | 7801790 |
|  | 11 | 3 and 6 and 10 | 6390 |
|  | 12 | 11 not ((case reports or case series or letter or correspondence).pt. or (case report* or case series or correspondence or conference or letter).ti,ab.) | 5669 |
|  | 13 | 12 not (exp Pediatrics/ or exp Child/ or exp Infant/ or exp Adolescent/ or pediatric*.ti,ab. or p*ediatric.ti,ab. or child*.ti,ab. or infant*.ti,ab. or adolescen*.ti,ab. or neonat*.ti,ab.) | 3536 |
|  | 14 | 13 not (exp Staphylococcus/ or staphylococc*.ti. or exp Streptococcus/ or streptococc*.ti. or exp Enterococcus/ or enterococc*.ti. or exp Salmonella/ or salmonella*.ti. or exp Mycobacterium/ or mycobacteri*.ti.) | 3197 |
|  | 15 | limit 14 to english language | 2993 |
|  | 16 | limit 15 to humans | 2748 |

| **Database** | **Number** | **Search strategy** | **Result, N,**  (31-Mar-2025) |
| --- | --- | --- | --- |
| **EMBASE(OVID)** | 1 | exp Bacteremia/ or exp Sepsis/ or (bloodstream infection* or bacteremia or septicemia).ti,ab,kw. | 397866 |
|  | 2 | exp Gram-Negative Bacterial Infections/ or exp Gram negative bacterial infection/ or exp Enterobacteriaceae/ or exp Enterobacterales/ or exp Pseudomonas Infections/ or exp Acinetobacter Infections/ or (Gram-negative bacill* or Gram-negative rod* or Enterobacteriaceae or Enterobacterale* or Escherichia coli or Klebsiella or Pseudomonas or Acinetobacter or Proteus or Enterobacter or Serratia).ti,ab,kw. | 1158299 |
|  | 3 | 1 and 2 | 66608 |
|  | 4 | exp Anti-Bacterial Agents/ or (antimicrobial* or antibiotic* or anti-infective*).ti,ab,kw. | 5336823 |
|  | 5 | (oral stepdown or switch therap* or oral antibiotic* or step-down therap*).ti,ab,kw. | 12731 |
|  | 6 | 4 or 5 | 6013227 |
|  | 7 | randomized controlled [trial.pt](http://trial.pt/). or randomized.ti,ab. or placebo.ti,ab. or clinical [trial.pt](http://trial.pt/). or (random* adj5 (assign* or allocat*)).ti,ab. | 1384187 |
|  | 8 | cohort [studies.pt](http://studies.pt/). or cohort*.ti,ab. or (follow-up or longitudinal or prospective or retrospective).ti,ab. or (incidence or risk or predict* or prognos*).ti,ab. | 10866598 |
|  | 9 | case-control [studies.pt](http://studies.pt/). or (case-control or case referent).ti,ab. or (retrospective adj3 (study or analysis)).ti,ab. or (odds ratio or matched or control group).ti,ab. | 2752879 |
|  | 10 | 7 or 8 or 9. | 12309819 |
|  | 11 | 3 and 6 and 10. | 20820 |
|  | 12 | 11 not ((case reports or case series or letter or correspondence).pt. or (case report* or case series or correspondence or conference or letter).ti,ab.) | 18714 |
|  | 13 | 12 not (exp Pediatrics/ or exp Child/ or exp Infant/ or exp Adolescent/ or pediatric.ti,ab. or pediatric.ti,ab. or child*.ti,ab. or infant*.ti,ab. or adolescen*.ti,ab. or neonat*.ti,ab.) | 13078 |
|  | 14 | 13 not (exp Staphylococcus/ or staphylococc*.ti. or exp Streptococcus/ or streptococc*.ti. or exp Enterococcus/ or enterococc*.ti. or exp Salmonella/ or salmonella*.ti. or exp Mycobacterium/ or mycobacteri*.ti.) | 6619 |
|  | 15 | limit 14 to (human and english language and yr="1980 -Current") | 5041 |

**Supplementary Table 2.** Articles excluded from the systematic review in order of publication year

| Study No. | Author, year | Reason for exclusion |
| --- | --- | --- |
| **Identification of studies in databases** | | |
| 1 | Paladino JA et al, 1991 | The study did not have detailed data on oral stepdown or intravenous antimicrobial therapy. |
| 2 | Endemiani A et al, 2004 | The study aim was not relevant (comparison of imipenem and ciprofloxacin efficacy against bloodstream infections due to ESBL-producing *Klebsiella pneumoniae).* |
| 3 | Montero JG et al, 2007 | The study aim was not relevant (comparison of initial combination versus single antimicrobial therapy for bloodstream infections due to *Pseudomonas aeruginosa.* |
| 4 | Vogelaers D et al, 2010 | The study aim was not relevant (patterns of empiric antimicrobial therapy for critically ill patients). |
| 5 | Esterly JS et al, 2011 | The study aim was not relevant (association between carbapenem resistance and antimicrobial therapy for bloodstream infections due to *Acinetobacter baumanii*). |
| 6 | Kim YJ et al, 2012 | The study aim was not relevant (risk factors of mortality in patients with a bloodstream infection due to carbapenem-resistant *Acinetobacter baumanii*). |
| 7 | Shime N et al, 2013 | The study aim was not relevant (comparison of de-escalation and non-de-escalation in the treatment of bloodstream infections due to enterobacterales). |
| 8 | Cho SY et al, 2014 | The study aim was not relevant (comparison of sulfamethoxazole/trimethoprim and levofloxacin in the treatment of bloodstream infections due to *Stenotrophomonas maltophilia*). |
| 9 | Falcone M et al, 2014 | The study aim was not relevant (risk factor analysis of bloodstream infections due to ESBL-producing enterobacterales). |
| 10 | Fitzpatrick JM et al, 2015 | The study aim was not relevant (association between empiric therapy and outcomes in bloodstream infection due to gram-negative bacteria). |
| 11 | Lee CC et al, 2015 | The study aim was not relevant (comparison of de-escalation and non-de-escalation in the treatment of bloodstream infections due to enterobacterales). |
| 12 | Patrick N et al, 2015 | Review article. |
| 13 | Kutob LF et al, 2016 | The study aim was not relevant (comparison of different oral stepdown antimicrobial therapies) |
| 14 | Veve MP et al, 2016 | The study aim was not relevant (comparison of intravenous fosfomycin and ertapenem). |
| 15 | Wang CH et al, 2016 | The study aim was not relevant (comparison of clinical outcomes of sulfamethoxazole/trimethoprim susceptible and non-susceptible *Stenotrophomonas maltophiliaI* causing bloodstream infections). |
| 16 | Lo CL et al, 2017 | The study aim was not relevant (comparison of carbapenem and fluoroquinolones in the treatment of bloodstream infections due to ESBL-producing enterobacterales). |
| 17 | Lopez-Cortes LE et al, 2017 | Trial protocol |
| 18 | Nelson AN et al, 2017 | The study aim was not relevant (comparison of short-course and long-course therapy for bloodstream infections due to gram-negative bacteria). |
| 19 | Uno S et al, 2017 | The study aim was not relevant (comparison of short-course and long-course therapy for bacteremic cholangitis). |
| 20 | Chotiprasitasakul D et al, 2018 | The study aim was not relevant (comparison of short-course and long-course therapy for bloodstream infections due to gram-negative bacteria). |
| 21 | Giannella M et al, 2018 | The study aim was not relevant (comparison of short-course and long-course therapy for bloodstream infections due to gram-negative bacteria). |
| 22 | Huang YC et al, 2018 | The study aim was not relevant (comparison of fluoroquinolone and non-fluoroquinolone in the treatment of bloodstream infections due to *Elizabethkingia meningoseptica.* |
| 23 | Ko JH et al, 2018 | The study aim was not relevant (comparison of non-carbapenems and carbapenems in the treatment of bloodstream infections due to ESBL-producing enterobacterales). |
| 24 | Mercuro NJ et al, 2018 | The study aim was not relevant (comparison of different oral stepdown antimicrobial therapies) |
| 25 | Palacios-Baena ZR et al, 2018 | The study aim was not relevant (comparison of de-escalation and non-de-escalation in the treatment of bloodstream infections due to enterobacterales). |
| 26 | Watson L et al, 2018 | The study aim was not relevant (comparison of sulfamethoxazole/trimethoprim and fluoroquinolones in the treatment of bloodstream infections due to *Stenotrophomonas maltophilia*). |
| 27 | Wu PF et al, 2018 | The study aim was not relevant (comparison of beta-lactam antimicrobials and fluoroquinolones [intravenous and oral] in the treatment of bloodstream infections due to *Pseudomonas aeruginosa*). |
| 28 | Fabre V et al, 2019 | The study aim was not relevant (comparison of short-course and long-course therapy for bloodstream infections due to *Pseudomonas aeruginosa*). |
| 29 | Kim SE et al, 2019 | The study aim was not relevant (comparison of third-generation cephalosporin plus ciprofloxacin and doxycycline therapy for *Vibrio vulnificus* septicemia). |
| 30 | Lee CC et al, 2019 | The study aim was not relevant (comparison of high-dose and conventional dose levofloxacin for bloodstream infections due to enterobacterales). |
| 31 | Sousa A et al, 2019 | The study aim was not relevant (comparison of short-course and long-course therapy for bloodstream infections due to gram-negative bacteria). |
| 32 | Yahav D et al, 2019 | The study aim was not relevant (comparison of short-course and long-course therapy for bloodstream infections due to gram-negative bacilli). |
| 33 | Al-Hasan MN et al, 2020 | The study had no detailed data on oral stepdown or intravenous antimicrobial therapy. |
| 34 | Amoah J et al, 2020 | Ad-hoc study (using the same database as Tamma, PD et al., 2019). |
| 35 | Claeys KC et al, 2020 | Antimicrobial stewardship program study without treatment or outcome information on individual patients. |
| 36 | Daneman N et al, 2020 | The study aim was not relevant (comparison of short-course and long course therapy for bloodstream infections). |
| 37 | Gunter SG et al, 2020 | The study aim was not relevant (comparison of fluoroquinolone and non-fluoroquinolone antimicrobials in the treatment of bloodstream infections due to chromosomally mediated AmpC-producing enterobacterales). |
| 38 | Hogan CA et al, 2020 | Antimicrobial stewardship program study (rapid antimicrobial susceptibility testing in diagnostic stewardship) |
| 39 | Nisly SA et al, 2020 | The study aim was not relevant (comparison of different oral stepdown antimicrobial therapies) |
| 40 | Ruigomez MR et al, 2020 | The study aim was not relevant (comparison of short-course and long-course therapy for bloodstream infections due to gram-negative bacteria). |
| 41 | Saad S et al, 2020 | The study aim was not relevant (comparison of different oral stepdown antimicrobial therapies) |
| 42 | Sutton JD et al, 2020 | The study aim was not relevant (comparison of different oral stepdown antimicrobial therapies) |
| 43 | Birrell MT et al, 2021 | Antimicrobial stewardship study (intervention for *E. coli* bloodstream infections without detailed individual patient data). |
| 44 | Chen L et al, 2021 | The study aim was not relevant (comparison of combination therapy and monotherapy for bloodstream infections due to carbapenem-resistant enterobacterales). |
| 45 | Kadri SS et al, 2021 | The study aim was not relevant (outcomes in patients with inappropriate empiric antibiotic therapy for bloodstream infections at U.S. hospitals based on discordant *In vitro s*usceptibilities). |
| 46 | Olsen J et al, 2021 | Pediatric study |
| 47 | Bae M et al, 2022 | The study aim was not relevant (comparison of short-course and long-course therapy for bloodstream infections due to *Pseudomonas aeruginosa*). |
| 48 | Babich T et al, 2022 | The study aim was not relevant (comparison of short-course and long course therapy for bloodstream infections due to *Pseudomonas aeruginosa*). |
| 49 | Dunne MW et al, 2022 | Unmatched target population (patients with pyelonephritis). |
| 50 | Eckburg PB et al, 2022 | Unmatched target population (patients with complicated urinary tract infection). |
| 51 | Gamble KC et al, 2022 | Unmatched target population (patients with no bloodstream infection). |
| 52 | Molina J et al, 2022 | The study aim was not relevant (comparison of short-course and long course therapy for bloodstream infections due to enterobacterales). |
| 53 | Sojo-Dorado J et al, 2022 | The study aim was not relevant (comparison of fosfomycin and comparators for bloodstream infections due to enterobacterales. Both arms included patients with oral stepdown therapy). |
| 54 | Tai T et al, 2022 | Antimicrobial stewardship program study (without treatment and outcome information of individual patients). |
| 55 | Alzaidi S et al, 2023 | The study aim was not relevant (comparison of different oral stepdown antimicrobial therapies) |
| 56 | Bjork L et al, 2023 | The study aim was not relevant (comparison of different oral stepdown antimicrobial therapies) |
| 57 | Mack T et al, 2023 | The study aim was not relevant (comparison of different oral stepdown antimicrobial therapies) |
| 58 | McAlister MJ et al, 2023 | The study aim was not relevant (comparison of different oral stepdown antimicrobial therapies) |
| 59 | Rodrigues RD et al, 2023 | The study aim was not relevant (comparison of short-course and long-course therapy for bloodstream infections due to *Pseudomonas aeruginosa* and *Acinetobacter baumanii*). |
| 60 | Sojo-Dorado J et al, 2023 | The study aim was not relevant (comparison of two different oral stepdown therapies) |
| 61 | Soto CL et al, 2023 | The study aim was not relevant (comparison of short-course and long-course therapy for bloodstream infections due to carbapenem-resistant enterobacterales). |
| 62 | Anderson DT et al, 2024 | The study aim was not relevant (comparison of short-course and long-course therapy for bloodstream infections due to gram-negative bacilli). |
| 63 | De la Villa S et al, 2024 | The study aim was not relevant (comparison of short-course and long-course therapy for bloodstream infections due to carbapenemase-producing enterobacterales). |
| 64 | Escrihuela-Vidal F et al, 2024 | The study aim was not relevant (comparison of de-escalation versus non-de-escalation in the treatment of bloodstream infections due to enterobacterales). |
| 65 | Feng X et al, 2024 | The study aim was not relevant (comparison of short-course and long-course therapy for bloodstream infections due to *Pseudomonas aeruginosa*). |
| 66 | Herrera F et al, 2024 | The study aim was not relevant (comparison of short-course and long-course therapy for bloodstream infections due to enterobacterales). |
| 67 | Lopez-Cortes LE et al, 2024 | The study aim was not relevant (comparison of de-escalation versus non-de-escalation in the treatment of bloodstream infections due to enterobacterales). |
| 68 | Miwa T et al, 2024 | The study aim was not relevant (comparison of short-course and long-course therapy for bloodstream infections due to gram-negative bacteria). |
| 69 | Mponponsuo K et al, 2024 | The study aim was not relevant (comparison of different oral stepdown antimicrobial therapies) |
| 70 | Tingsgård S et al, 2024 | The study aim was not relevant (comparison of short-course and long-course therapy for bloodstream infections due to gram-negative bacteria). |
| 71 | You TY et al, 2024 | The study aim was not relevant (comparison of short-course and long-course therapy for bloodstream infections due to *Klebsiella pneumoniae*). |
| 72 | Hasegawa K et al, 2025 | The study aim was not relevant (although the study compared intravenous therapy with oral stepdown therapy, the oral therapy group included persons only receiving oral antimicrobial therapy). |
| **Identification via other methods** | | |
| 73 | Peacock JE et al, 1989 | The study included bloodstream infections due to gram-positive organisms. |
| 74 | Groton MA et al, 1996 | The study included bloodstream infections due to gram-positive organisms. |
| 75 | Park TY et al. 2014 | The study included bloodstream infections due to gram-positive and polymicrobial organisms |
| 76 | Sessa J et al, 2018 | The study aim was not relevant (comparison of oral stepdown therapy with fluoroquinolone and sulfamethoxazole/trimethoprim). |
| 77 | Panjubi C et al, 2019 | Review article |
| 78 | Al-Hasan MN et al, 2020 | Review article |
| 79 | Lee CC et al, 2020 | The study included bloodstream infections due to gram-positive organisms. |
| 80 | Utley S et al, 2020 | Not a comparative study |
| 81 | Hansen BÅ 2022 | Not a comparative study |
| 82 | Nussbaum EZ et al, 2023 | Duplication of an included study (Nussbaum EZ et al, 2024). |
| 83 | Omrani AS et al, 2024 | Duplication of an included study (Omrani AS et al, 2024) |
| 84 | Li Q eat al, 2025 | Review article |

**Supplementary Table 3.** The Preferred Reporting Items for Systematic reviews and Meta-Analyses (PRIMSA) statement checklist

| **Section and Topic** | **Item #** | **Checklist item** | **Location where item is reported** |
| --- | --- | --- | --- |
| **TITLE** | | |  |
| Title | 1 | Identify the report as a systematic review. | 1 |
| **ABSTRACT** | | |  |
| Abstract | 2 | See the PRISMA 2020 for Abstracts checklist. | 3-4 |
| **INTRODUCTION** | | |  |
| Rationale | 3 | Describe the rationale for the review in the context of existing knowledge. | 5 |
| Objectives | 4 | Provide an explicit statement of the objective(s) or question(s) the review addresses. | 5 |
| **METHODS** | | |  |
| Eligibility criteria | 5 | Specify the inclusion and exclusion criteria for the review and how studies were grouped for the syntheses. | 6-7 |
| Information sources | 6 | Specify all databases, registers, websites, organisations, reference lists and other sources searched or consulted to identify studies. Specify the date when each source was last searched or consulted. | 6 |
| Search strategy | 7 | Present the full search strategies for all databases, registers and websites, including any filters and limits used. | 6 Supplementary table 1. |
| Selection process | 8 | Specify the methods used to decide whether a study met the inclusion criteria of the review, including how many reviewers screened each record and each report retrieved, whether they worked independently, and if applicable, details of automation tools used in the process. | 6-7 |
| Data collection process | 9 | Specify the methods used to collect data from reports, including how many reviewers collected data from each report, whether they worked independently, any processes for obtaining or confirming data from study investigators, and if applicable, details of automation tools used in the process. | 7-8 |
| Data items | 10a | List and define all outcomes for which data were sought. Specify whether all results that were compatible with each outcome domain in each study were sought (e.g. for all measures, time points, analyses), and if not, the methods used to decide which results to collect. | 7-8 |
|  | 10b | List and define all other variables for which data were sought (e.g. participant and intervention characteristics, funding sources). Describe any assumptions made about any missing or unclear information. | Table 1 and supplementary table 4. |
| Study risk of bias assessment | 11 | Specify the methods used to assess risk of bias in the included studies, including details of the tool(s) used, how many reviewers assessed each study and whether they worked independently, and if applicable, details of automation tools used in the process. | 7-8 |
| Effect measures | 12 | Specify for each outcome the effect measure(s) (e.g. risk ratio, mean difference) used in the synthesis or presentation of results. | 8-9 |
| Synthesis methods | 13a | Describe the processes used to decide which studies were eligible for each synthesis (e.g. tabulating the study intervention characteristics and comparing against the planned groups for each synthesis (item #5)). | 8-9 |
|  | 13b | Describe any methods required to prepare the data for presentation or synthesis, such as handling of missing summary statistics, or data conversions. | 7-9 |
|  | 13c | Describe any methods used to tabulate or visually display results of individual studies and syntheses. | 8-9 |
|  | 13d | Describe any methods used to synthesize results and provide a rationale for the choice(s). If meta-analysis was performed, describe the model(s), method(s) to identify the presence and extent of statistical heterogeneity, and software package(s) used. | 8-9 |
|  | 13e | Describe any methods used to explore possible causes of heterogeneity among study results (e.g. subgroup analysis, meta-regression). | 9 |
|  | 13f | Describe any sensitivity analyses conducted to assess robustness of the synthesized results. | 9 |
| Reporting bias assessment | 14 | Describe any methods used to assess risk of bias due to missing results in a synthesis (arising from reporting biases). | 7-8 |
| Certainty assessment | 15 | Describe any methods used to assess certainty (or confidence) in the body of evidence for an outcome. | 8 |
| **RESULTS** | | |  |
| Study selection | 16a | Describe the results of the search and selection process, from the number of records identified in the search to the number of studies included in the review, ideally using a flow diagram. | 10 |
|  | 16b | Cite studies that might appear to meet the inclusion criteria, but which were excluded, and explain why they were excluded. | 10  Supplementary table 2 |
| Study characteristics | 17 | Cite each included study and present its characteristics. | 11 Supplementary table 4 |
| Risk of bias in studies | 18 | Present assessments of risk of bias for each included study. | 12  Supplementary figure 3 |
| Results of individual studies | 19 | For all outcomes, present, for each study: (a) summary statistics for each group (where appropriate) and (b) an effect estimate and its precision (e.g. confidence/credible interval), ideally using structured tables or plots. | 13-15 |
| Results of syntheses | 20a | For each synthesis, briefly summarise the characteristics and risk of bias among contributing studies. | 12-13 Supplementary figure 4. |
|  | 20b | Present results of all statistical syntheses conducted. If meta-analysis was done, present for each the summary estimate and its precision (e.g. confidence/credible interval) and measures of statistical heterogeneity. If comparing groups, describe the direction of the effect. | 12-13 Supplementary figure 3 |
|  | 20c | Present results of all investigations of possible causes of heterogeneity among study results. | 12-13 Supplementary figure 4 |
|  | 20d | Present results of all sensitivity analyses conducted to assess the robustness of the synthesized results. | 12-13, FIgure 1 and 2. |
| Reporting biases | 21 | Present assessments of risk of bias due to missing results (arising from reporting biases) for each synthesis assessed. | 11 Supplementary figure 2 |
| Certainty of evidence | 22 | Present assessments of certainty (or confidence) in the body of evidence for each outcome assessed. | 12-13 |
| **DISCUSSION** | | |  |
| Discussion | 23a | Provide a general interpretation of the results in the context of other evidence. | 14-15 |
|  | 23b | Discuss any limitations of the evidence included in the review. | 16 |
|  | 23c | Discuss any limitations of the review processes used. | 16 |
|  | 23d | Discuss implications of the results for practice, policy, and future research. | 16 |
| **OTHER INFORMATION** | | |  |
| Registration and protocol | 24a | Provide registration information for the review, including register name and registration number, or state that the review was not registered. | 6, 17 |
|  | 24b | Indicate where the review protocol can be accessed, or state that a protocol was not prepared. | 17 |
|  | 24c | Describe and explain any amendments to information provided at registration or in the protocol. | 17 |
| Support | 25 | Describe sources of financial or non-financial support for the review, and the role of the funders or sponsors in the review. | 17 |
| Competing interests | 26 | Declare any competing interests of review authors. | 17 |

**Supplementary Table 4.** Characteristics of studies included in the systematic review and meta-analysis

| **First author, year, country** | **Study design**  **(total sample size)** | **Patient population** | **Sources of BSI in oral stepdown therapy cohort (%)** | **Sources of BSI in IV therapy cohort (%)** | **Oral stepdown therapy types (%)** | **Definition of oral stepdown therapy** | **Differences in demographic and clinical characteristics between the group** |
| --- | --- | --- | --- | --- | --- | --- | --- |
|  |  |  | **Causative pathogens (%)** | **Causative pathogens (%)** |  |  |  |
| Meije Y  2019  Spain | Monocentric retrospective cohort study (N=96) | Mixed population | Urinary 71.4%  Biliary 7.1% CRBSI 9.5% | Urinary 57.6%  Biliary 20.3%  CRBSI 3.4% | ST 67.6% Quinolones 24.3% Fosfomycin 5.4% Amox/Clav 2.7% | The timing of the transition to oral stepdown therapy was not specified. The median duration to the transition was 2.5 days (IQR: 0, 6). | Higher prevalence of BSI due to Hepatobiliary infection in the IVAT group.  Higher Pitt bacteremia score in the IVAT group. |
|  |  |  | ESBL- or AmpC-producing  *E. coli* or *K. pneumoniae* (100%) | ESBL- or AmpC-producing  *E. coli* or *K. pneumoniae* (100%) |  |  |  |
| Tossey JC  2021  USA | Monocentric retrospective cohort study (N=211) | Immuno-compromised population only | CRBSI 38.5% Urinary 27.5%  LCBI 20.5% IAI 6.4% | CRBSI 42.9%  Urinary 24.1%  LCBI 9.0%  IAI 9.0% | Ciprofloxacin 96.0%  Levofloxacin 4.0% | The timing of the transition to oral stepdown therapy was not specified. The median duration to the transition was 7.0 days (IQR: 5.8, 9.4). | Higher Pitt bacteremia score in the IVAT group.  Higher proportion of neutropenia, hematologic malignancies, and ICU admission in the IVAT group. |
|  |  |  | *K. pneumoniae* 37.2% *E. coli* 35.9% *P. aeruginosa* 5.1% | *K. pneumoniae* 36.1% *E.coli* 26.3% *P. aeruginosa* 17.3% |  |  |  |
| Tingsgård S 2024  Denmark | Multicentric retrospective cohort study with target trial emulation framework (N=914) | Mixed population | Urinary 76.5%  GI 17.1%  GI surgery 1.8% | Urinary 76.5%  GI 17.1% GI surgery 1.8% | Beta-lactams 63.1%  Ciprofloxacin 16.6%  ST 0.4% | The transition from IV to oral stepdown therapy was less than 4 days. | Higher proportion of younger population and fewer ICU admission in the OSAT group. |
|  |  |  | *E. coli* 79.4% *Klebsiella* spp. 9.5% *Enterobacter* spp. 2.8%  *Proteus* spp. 2.3% | *E. coli* 72.6% *Klebsiella* spp. 15.2% *Enterobacter* spp. 3.3%  *Proteus* spp. 4.2% |  |  |  |
| Engers DW 2024  USA | Multicentric retrospective cohort study (N=4,581) | Mixed population | Urinary 64.9%  Biliary 12.1% IAI 9.9% | Urinary 38.6%  IAI 16.4% Biliary 11.1% CRBSI 9.3% | Quinolones 62.2% Beta-lactams 28.3%  ST 11.5% | The transition from IV to oral stepdown therapy was less than 7 days. The median duration to the transition was 5 days (IQR: 4, 6). | Patients with IVAT had higher severity of GNB-BSI (higher proportion of ICU admission, higher Pitt bacteremia score, more comorbid illnesses). |
|  |  |  | *E. coli* 60.4% *Klebsiella* spp.18.8% *Enterobacter* spp.4.1%  *Proteus* spp. 3.9% | *E. coli* 44.9% *Klebsiella* spp. 20.9% *Enterobacter* spp. 6.4%  *Proteus* spp. 6.1% |  |  |  |
| Rieger KL  2017  USA | Monocentric retrospective cohort study (N=241) | General population only | Urinary 100% | Urinary 100% | Quinolones 65.3%  Beta-lactams 19%  ST 9.1% | The timing of the transition to oral step-down therapy was not specified. The median duration to the transition was 4 days (IQR: 2, 5). | Patients with IVAT had more comorbid illnesses (higher Charlson comorbidity index). |
|  |  |  | *E. coli* 62% | *E. coli* 50% |  |  |  |
| Pradubkham T 2021  Thailand | Monocentric retrospective cohort study (N=955) | Mixed population | Primary 20.2%  Urinary 41.5%  IAI 29.0% Pulmonary 6.6% | Primary 24.6%  Urinary 35.4% IAI 22.0% Pulmonary 5.6% | Ciprofloxacin 53.0% Cefixime 20.9% Amox/Clav 10.3% Cefdinir 9.9% | The timing of the transition to oral step-down therapy was not specified. The median duration to the transition was 6 days (IQR: 4, 7). | Similar characteristics  between two groups after propensity score matching. |
|  |  |  | *E. coli* 60.1% *Klebsiella* spp.14.1% *P. aeruginosa* 4.6% *Proteus* spp. 3.0% *Enterobacter* spp.2.1% | *E. coli* 58.0% *Klebsiella* spp.18.0% *P. aeruginosa* 4.3% *Proteus* spp. 3.0% *Enterobacter* spp.3.0% |  |  |  |
| Avila-Nunez M 2023  Spain | Monocentric retrospective cohort study (N=77) | General population only | Urinary 38.9%  CRBSI 11.1%  IAI 5.6% | Urinary 29.3% CRBSI 17.1%  IAI 14.6% | ST 57.0% Quinolones 35.0% | The timing of the transition to oral step-down therapy was not specified. The duration was unclear. | Only included GNB=BSI due to AmpC betalactamase producing organisms.  Higher proportion of sepsis in the IVAT group. |
|  |  |  | AmpC-producing enterobacterales 100% | AmpC-producing enterobacterales 100% |  |  |  |
| Noguchi T  2022  Japan | Multicentric retrospective cohort study (N=73) | General population only | Urinary 100% | Urinary 100% | ST 50.0%  Levofloxacin 27.0% Ciprofloxacin 12.0% Fosfomycin 8.0% Minocycline 4.0% | The timing of the transition from IV to oral stepdown therapy was less than 10 days. The median duration to the transition was 7 days (IQR: 4, 8.75). | No statistical differences in clinical characteristics between two groups.  Only included GNB=BSI due to ESBL-producing organisms. |
|  |  |  | ESBL *E.coli* 100% | ESBL *E.coli* 100% |  |  |  |
| Omrani AS  2024  Qatar | Multicentric* open-label, non-inferiority RCT (N=164) | General population only | Urinary 60.6% IAI 9.0% Biliary 9.0% Primary 7.9% | Urinary 60.0% IAI 16.5%  Biliary 4.7% Primary 9.4% | Cephalosporin 34.8%  BL/BLI 30.3%  Quinolones 19.1%  ST 15.7% | The transition to oral stepdown therapy followed the completion of 3 - 5 days of active IV therapy. The median duration to the transition was 4 days (IQR: 3, 5). | No substantial differences in clinical characteristics between two groups. |
|  |  |  | *E. coli* 68.5%  *Klebsiella* spp. 22.5% *Enterobacter* spp.6.7% | *E. coli* 64.7%  *Klebsiella* spp. 25.9% *Enterobacter* spp.7.1% |  |  |  |
| Tamma PD  2019  USA | Multicentric retrospective cohort study (N=1,478) | Mixed population | Urinary 39.9% IAI 20.6% Biliary 13.9% CRBSI 18.3% | Urinary 40.5% IAI 19.6% Biliary 14.5% CRBSI 18.5% | Ciprofloxacin 45.6% Levofloxacin 23.1%  ST 13.4%  Amox/Clav 5.1% Cefidinir 4.1%  Cefixime 2.8% Cefpodoxime 2.3%  Cephalexin 2.2% | The transition to oral stepdown therapy was made within 4 days of the completion of IV therapy. The median duration to the transition was 3 days (IQR: 2, 4). | Similar characteristics between two groups after propensity score matching |
|  |  |  | *E.coli* 41.8% *Klebsiella* spp. 37.6% *Enterobacter* spp.11.1%  *Proteus* spp. 5.4% | *E.coli* 45.5% *Klebsiella* spp. 33.7% *Enterobacter* spp. 12.3%  *Proteus* spp. 3.4% |  |  |  |
| Nguyen N  2023  USA | Monocentric retrospective cohort study (N=199) | Unknown | Urinary 51.8% IAI 22.4%  Other 25.9% | Urinary 57.0% IAI 20.2% Other 22.8% | Cefdinir 47.1%  Cephalexin 20.7%  Levofloxacin 8.0%  Ciprofloxacin 5.7%  Amox/Clav 5.7%  Cefuroxime 4.6%  Cefpodoxime 4.6%  ST 2.3% | The timing of the transition to oral step-down therapy was not specified. The median duration to the transition was 3 days (IQR: 2, 5). | Patients with IVAT had higher Charlson comorbidity index score, higher proportion of ICU admission, and higher prevalence of GNB-BSI due to MDROs. |
|  |  |  | *E. coli* 62.4%  *K. pneumoniae*15.3%  Other 22.3% | *E. coli* 53.5%  *K. pneumoniae*16.7%  Other 29.8% |  |  |  |
| Veillette JJ  2024  USA | Multicentric^#^ retrospective cohort study (N=759) | Mixed population | Urinary 100% | Urinary 100% | Quinolones 44.4%  HBBL 32.9%  LBBL 11.5%  ST 11.2% | The duration of the transition from IV to oral stepdown therapy was less than 7 days. The median duration to the transition was 3 days (IQR: 3, 4). | Patients with IVAT had more comorbidities, urologic abnormalities, and ESBL-producing isolates, however, the proportion of ICU admission and clinical stability in 3 days was similar between two groups. |
|  |  |  | *E. coli* 82.0%  *K. pneumoniae* 18.0% | *E. coli* 81.5%  *K. pneumoniae* 18.5% |  |  |  |
| Nussbaum EZ 2024  USA | Multicentric retrospective cohort study (N=162) | Immuno-compromised population only | Urinary 59.2% Biliary 11.7%  IAI 8.3%  SSTI 4.2% | Urinary 78.6% Biliary 2.4% IAI 7.1%  SSTI 0% | Ciprofloxacin 68.3%  Levofloxacin 18.3%  Amox/Clav 5.0%  ST 4.2% | The timing of the transition to oral step-down therapy was not specified. The median duration to the transition was 4 days (IQR: 1, 15). | Only included patients with solid organ transplanted patients.  Clinical characteristics between two groups were similar. |
|  |  |  | *E. coli* 40.0%  *Klebsiella spp.* 25.8%  *Enterobacter* spp. 13.3%  *Pseudomonas* spp. 3.3% | *E. coli* 61.9%  *Klebsiella* spp. 16.7%  *Enterobacter* spp. 7.1%  *Pseudomonas* spp. 2.4% |  |  |  |
| Savage H  2021  USA | Monocentric retrospective cohort study (N=31) | Immuno-compromised population only | NS | NS | NS | The timing of the transition to oral step-down therapy was not specified. The median duration to the transition was unclear. | Detailed data were unavailable because of a conference abstract |
|  |  |  | NA | NA |  |  |  |
| Mulvey N  2021  USA | Monocentric, retrospective cohort study (N=130) | Unknown | Urinary 100% | Urinary 100% | NS | The timing of the transition to oral step-down therapy was not specified. The median duration to the transition was unclear. | Detailed data were unavailable because of a conference abstract |
|  |  |  | NA | NA |  |  |  |
| Williams K  2019  USA | Monocentric, retrospective cohort study (N=99) | Unknown | NS | NS | NS | The timing of the transition to oral step-down therapy was not specified. The median duration to the transition was unclear. | Patients with IVAT had higher Pitt bacteremia score. |
|  |  |  | NA | NA |  |  |  |

**NOTE.** BSI, bloodstream infection; IV, intravenous; CRBSI, catheter-related bloodstream infection; LCBI, laboratory-confirmed bloodstream infection; IAI, intra-abdominal infection; GI, gastrointestinal; SSTI, skin and soft tissue infection; NS, not stated; ESBL, extended-spectrum beta lactamase; ST, sulfamethoxazole-trimethoprim; Amox/Clav, amoxicillin/clavulanate; BL/BLI, beta-lactam/beta-lactamase inhibitor; HBBL, high bioavailability beta-lactams; LBBL, low bioavailability beta-lactams; IQR, interquartile range; *K. pneumoniae*, *Klebsiella pneumoniae; E.coli, Escherichia coli; P. aeruginosa, Pseudomonas aeruginosa.*

*Study institutions included hospitals in Bahrain, Kuwait, Qatar, and Türkiye.

# Study institutions included 23 hospitals and emergency departments (EDs), 38 urgent cares, and 300 primary care clinics.

**Supplementary Table 5.** Crude mortality rate, treatment failure, and length of hospitalization in the studies included in the systematic review and meta-analysis

| **First author, year** | **Treatment group** | **Mortality %, (N)** | **Treatment failure %, (N)** | **Length of stay median, (IQR)** |
| --- | --- | --- | --- | --- |
| Avila-Nunez M (2023) | PO step-down  IV | NA | 2.8% (1/36)  4.9% (2/41) | 14 (7, 22)  18 (13, 38) |
| Engers DW  (2024) | PO step-down  IV | NA | 16.2% (319/1969)  21.0% (549/2612) | 4 (3, 6)  12 (7, 24) |
| Meije Y  (2019) | PO step-down  IV | 5.4% (2/37)  10.2% (6/59) | 5.4% (2/37)  15.3% (9/59) | 7 (5, 10)  12 (9, 18) |
| Mulvey N  (2021) | PO step-down  IV | NA | 11.4% (10/88)  16.7% (7/42) | NA |
| Nguyen N (2023) | PO step-down  IV | 4.7% (4/85)  26.3% (30/114) | 0% (0/85)  1.8% (2/114) | 5 (3, 7)  5 (3, 10.8) |
| Noguchi T (2022) | PO step-down  IV | 3.8% (1/26)  2.1% (1/47) | 11.5% (3/26)  8.5% (4/47) | 13.5 (10, 32.75)  20 (15, 31) |
| Nussbaum (2024) | PO step-down  IV | 0% (0/120)  0% (0/42) | 0% (2/120)  0% (2/42) | 5 (range: 3-16)  7 (range: 4-18) |
| Omrani AS (2024) | PO step-down  IV | 7.9% (7/89)  7.1% (6/85) | 16.9% (15/89)  21.2% (18/85) | 6 (5, 8)  9 (6, 14) |
| Pradubkham T (2021) | PO step-down  IV | 0.3% (1/297)  1.7% (5/297) | 6.7% (20/297)  3.0% (9/297) | 7 (5, 10)  14 (9, 21) |
| Rieger KL (2017) | PO step-down  IV | 2.2% (3/135)  14.2% (15/106) | 8.1% (11/135)  3.8% (4/106) | 4.6 (3.1, 7.8)  7.1 (4.0, 17.5) |
| Savage H (2021) | PO step-down  IV | NA | 15.4% (4/26)  20.0% (1/5) | 7.4 (IQR NA)  13.6 (IQR NA) |
| Tamma PD (2019) | PO step-down  IV | 13.1% (97/739)  13.4% (99/739) | 0.5% (4/739)  0.8% (6/739) | 5 (3, 8)  7 (4, 14) |
| Tingsgård S (2024) | PO step-down  IV | 6.9% (30/433)  14.3% (69/481) | NA | NA |
| Tossey JC (2021) | PO step-down  IV | 1.3% (1/78)  7.5% (10/133) | 7.7% (6/78)  16.5% (22/133) | 7 (5, 14)  18 (12, 24) |
| Veillette JJ (2024) | PO step-down  IV | 1.7% (11/651)  1.9% (2/108) | 13.7% (89/651)  20.4% (22/108) | 69-72 hours (IQR NA)  91 hours (IQR NA) |
| Williams K  (2019) | PO step-down  IV | NA | 18.4% (9/49)  52.0% (26/50) | NA |

**ABBREVIATIONS.** PO, per os; IV, intravenous; NA, not available; IQR, interquartile range (Word count for Table 3 was 317).

**Supplementary Table 6.** Summary of the pooled results of the subgroup analyses

| **Outcomes (n)** | **Oral stepdown therapy** | **Intravenous therapy** | **Risk ratio or risk difference (95% CI)** | **I^2^** | **Overall effect**  **P value** |
| --- | --- | --- | --- | --- | --- |
| **Subgroup analysis stratified by studies including immunocompromised patients** | | | | | |
| Mortality (n=7) | 142/2,277 (6.2%) | 191/1,937 (9.9%) | 0.60 (0.33, 1.11) | 59% | 0.09 |
| Treatment failure (n=8) | 428/3,740 (11.4%) | 618/3,740 (16.5%) | 0.74 (0.66, 0.84) | 0% | <0.001 |
| **Subgroup analysis stratified by studies only limited to drug-resistant pathogens (AmpC or ESBL producing GNB)** | | | | | |
| Mortality (n=2) | 3/63 (4.8%) | 7/106 (6.6%) | 0.72 (0, 564.30) | 0% | 0.64 |
| Treatment failure (n=3) | 6/99 (6.1%) | 15/147 (10.2%) | 0.69 (0.10, 4.54) | 0% | 0.48 |
| **Subgroup analysis stratified by studies with oral transition within 5 days (median or mean)** | | | | | |
| Mortality (n=8) | 154/2289 (9.0%) | 227/1734 (12.9%) | 0.60 (0.25, 1.07) | 75% | 0.07 |
| Treatment failure (n=8) | 442/3825 (3.0%) | 612/3865 (3.8%) | 0.76 (0.67, 0.87) | 0% | 0.002 |
| **Subgroup analysis stratified by studies with 30-day mortality** | | | | | |
| Mortality (n=4) | 99/981 (10.1%) | 107/954 (11.2%) | 0.95 (0.61, 1.49) | 0% | 0.70 |
| Treatment failure (n=4) | 8/981 (0.9%) | 19/954 (2.0%) | 0.45 (0.24,0.85) | 0% | 0.03 |
| **Subgroup analysis stratified by studies only focusing on bacteremia originating from urinary tract infection** | | | | | |
| Mortality (n=4) | 15/812 (1.8%) | 18/261 (6.9%) | 0.49 (0.02, 10.91) | 57% | 0.43 |
| Treatment failure (n=4) | 113/900 (12.6%) | 37/303 (12.2%) | 0.88 (0.38, 2.03) | 33% | 0.67 |

**ABBREVIATION**. CI, confidence interval; ESBL, extended-spectrum beta-lactamase; GNB, gram-negative bacilli

**Supplementary Table 7.** Grading of Recommendations Assessment, Development and Evaluation (GRADE) for subgroup analyses

| **Certainty assessment** | | | | | | | | **№ of patients** | | **Effect** | | **Certainty** | **Importance** |
| --- | --- | --- | --- | --- | --- | --- | --- | --- | --- | --- | --- | --- | --- |
| **№ of studies** | **Study design** | | **Risk of bias** | **Inconsistency** | **Indirectness** | **Imprecision** | **Other considerations** | **Oral stepdown therapy** | **Intravenous therapy** | **Relative (95% CI)** | **Absolute (95% CI)** |  |  |
| **Mortality (Including immunocompromised patients)** | | | | | | | | | | | | | |
| 7 | Non-randomized studies | | Very serious^a^ | Not serious | Not serious | Not serious | All plausible residual confounding would suggest spurious effect, while no effect was observed | 142/2277 (6.2%) | 191/1937 (9.9%) | **RR 0.60** (0.33 to 1.11) | **39 fewer per 1000** (from 66 fewer to 11 more) | ⨁◯◯◯ Very low^a^ | CRITICAL |
| **Treatment failure (Including immunocompromised patients)** | | | | | | | | | | | | | |
| 8 | Non-randomized studies | | Very serious^a^ | Not serious | Not serious | Not serious | All plausible residual confounding would suggest spurious effect, while no effect was observed | 428/3740 (11.4%) | 618/3740 (16.5%) | **RR 0.74** (0.66 to 0.84) | **43 fewer per 1000** (from 56 fewer to 26 fewer) | ⨁◯◯◯ Very low^a^ | CRITICAL |
| **Mortality (patients with bacteremic urinary tract infection only)** | | | | | | | | | | | | | |
| 4 | Non-randomized studies | Very serious^a^ | | Not serious | Not serious | Serious^b^ | All plausible residual confounding would suggest spurious effect, while no effect was observed | 15/812 (1.8%) | 18/261 (6.9%) | **RR 0.49** (0.02 to 10.91) | **35 fewer per 1000** (from 68 fewer to 683 more) | ⨁◯◯◯ Very low^a,b^ | CRTICAL |
| **Treatment failure (patients with bacteremic urinary tract infection only)** | | | | | | | | | | | | | |
| 4 | Non-randomized studies | Very serious^a^ | | Serious^c^ | Not serious | Not serious | All plausible residual confounding would suggest spurious effect, while no effect was observed | 113/900 (12.6%) | 37/303 (12.2%) | **RR 0.88** (0.38 to 2.04) | **15 fewer per 1000** (from 76 fewer to 127 more) | ⨁◯◯◯ Very low^a,c^ | CRTICAL |

| **Mortality (Patients with GNB-BSI due to multidrug-resistant organisms [AmpC or ESBL producing organisms])** | | | | | | | | | | | | |
| --- | --- | --- | --- | --- | --- | --- | --- | --- | --- | --- | --- | --- |
| 2 | Non-randomised studies | Very serious | Not serious | Not serious | Not serious | All plausible residual confounding would suggest spurious effect, while no effect was observed | 3/63 (4.8%) | 7/106 (6.6%) | **RR 0.72** (0.00 to 564.30) | **18 fewer per 1000** (from -- to 1000 more) | ⨁◯◯◯ Very low | CRITICAL |
| **Treatment failure (Patients with GNB-BSI due to multidrug-resistant organisms [AmpC or ESBL producing organisms])** | | | | | | | | | | | | |
| 3 | Non-randomised studies | Very serious | Not serious | Not serious | Not serious | All plausible residual confounding would suggest spurious effect, while no effect was observed | 6/99 (6.1%) | 15/147 (10.2%) | **RR 0.69** (0.10 to 4.54) | **32 fewer per 1000** (from 92 fewer to 361 more) | ⨁◯◯◯ Very low | CRITICAL |

| **Mortality (patients with transitioning to OSAT within 5 days [median or mean])** | | | | | | | | | | | | |
| --- | --- | --- | --- | --- | --- | --- | --- | --- | --- | --- | --- | --- |
| 7 | Non-randomised studies | Very serious | Not serious | Not serious | Not serious | All plausible residual confounding would suggest spurious effect, while no effect was observed | 147/2200 (6.7%) | 221/1649 (13.1%) | **RR 0.46** (0.25 to 1.03) | **72 fewer per 1000** (from 107 fewer to 4 more) | ⨁◯◯◯ Very low | CRITICAL |
| **Treatment failure (patients with transitioning to OSAT within 5 days [median or mean])** | | | | | | | | | | | | |
| 7 | Non-randomised studies | Very serious | Not serious | Not serious | Not serious | all plausible residual confounding would suggest spurious effect, while no effect was observed | 427/3736 (11.4%) | 594/3780 (15.7%) | **RR 0.76** (0.66 to 0.88) | **38 fewer per 1000** (from 53 fewer to 19 fewer) | ⨁◯◯◯ Very low | CRITICAL |
| **Mortality (patients in the studies with 30-days mortality)** | | | | | | | | | | | | |
| 4 | Non-randomised studies | Very serious | Not serious | Not serious | Not serious | all plausible residual confounding would suggest spurious effect, while no effect was observed | 99/981 (10.1%) | 107/954 (11.2%) | **RR 0.95** (0.61 to 1.49) | **6 fewer per 1000** (from 44 fewer to 55 more) | ⨁◯◯◯ Very low | CRITICAL |
| **Treatment failure (patients in the studies with 30-days mortality)** | | | | | | | | | | | | |
| 4 | Non-randomised studies | Very serious | Not serious | Not serious | Not serious | all plausible residual confounding would suggest spurious effect, while no effect was observed | 8/981 (0.8%) | 19/954 (2.0%) | **RR 0.45** (0.24 to 0.85) | **11 fewer per 1000** (from 15 fewer to 3 fewer) | ⨁◯◯◯ Very low | CRITICAL |

**NOTE.** ^a^ Risk of bias was downgraded because of a lack of adjusting for potential confounders.

^b^ Imprecision was downgraded because fewer events occurred in both groups. ^c^ Inconsistency was

downgraded because a variation in results was observed across the studies.

**Supplementary Figure 1.** The Preferred Reporting Items for Systematic reviews and Meta-Analyses (PRIMSA) flow diagram for the present systematic review, which included searches

of databases, registers, and other sources

**
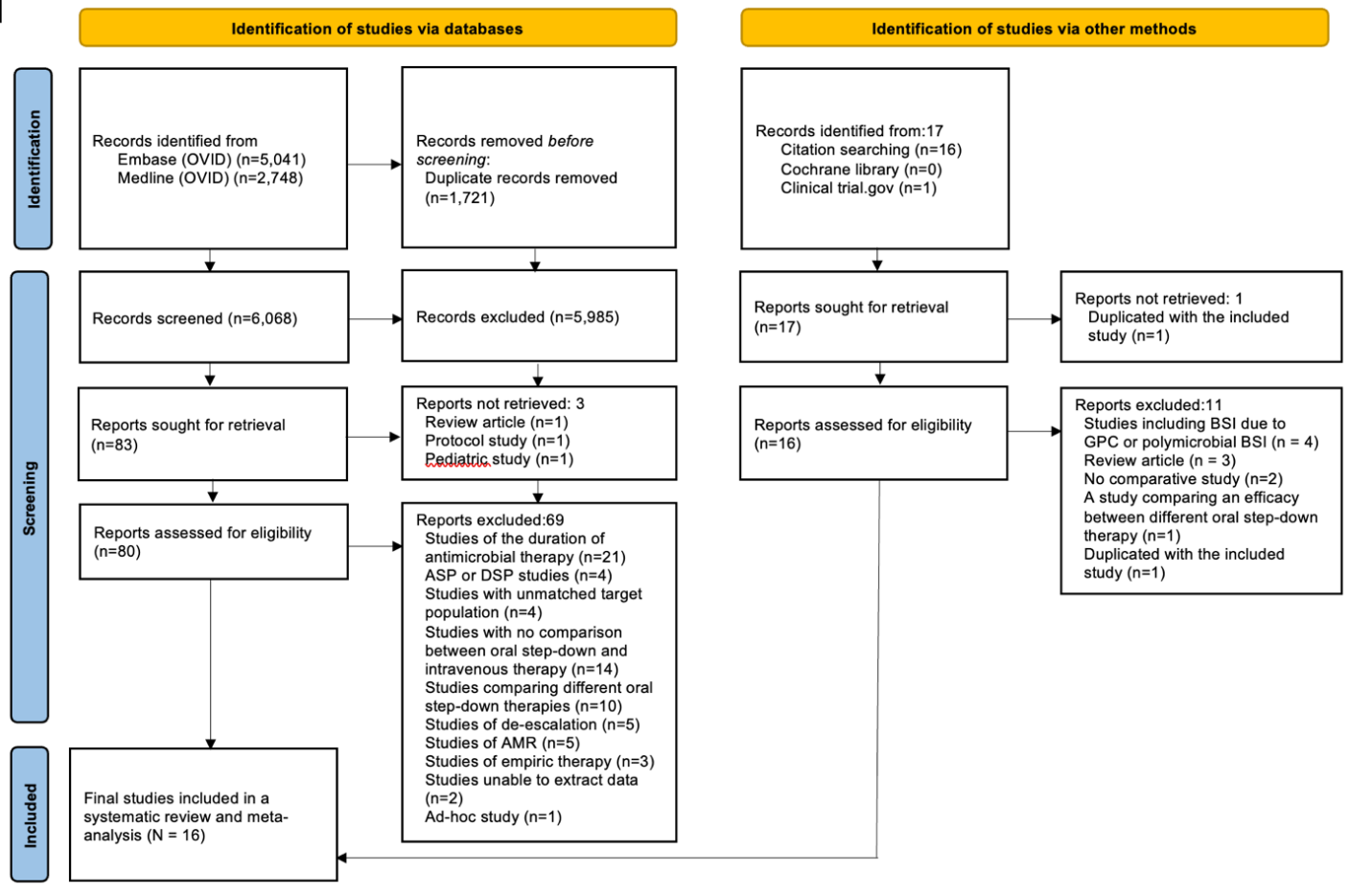
**

**Supplementary Figure 2.** Funnel plots of the sensitivity (expanded) analysis

**Supplementary Figure 2A**. A funnel plot of the mortality rate in 11 studies with 10 effect estimates in the sensitivity analysis

**
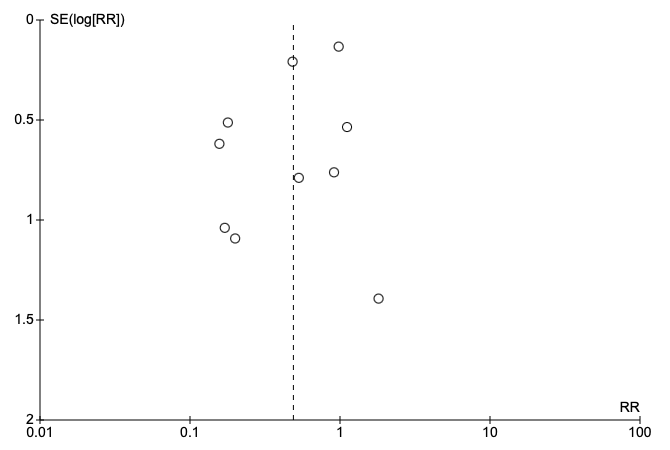
**

**NOTE.** The effect estimate in one study (Nussubaum, et al) was not included in the funnel plot because the number of deaths in both cohort was 0.

**Supplementary Figure 2B.** A funnel plot of the treatment failure rate in 15 studies with 15 effect estimates in the sensitivity analysis


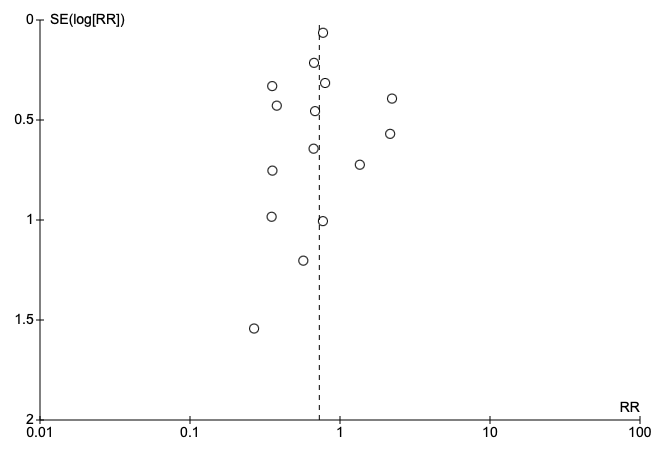


**Supplementary Figure 3.** Risk of bias of the studies included in the meta-analysis

**[Observational study]
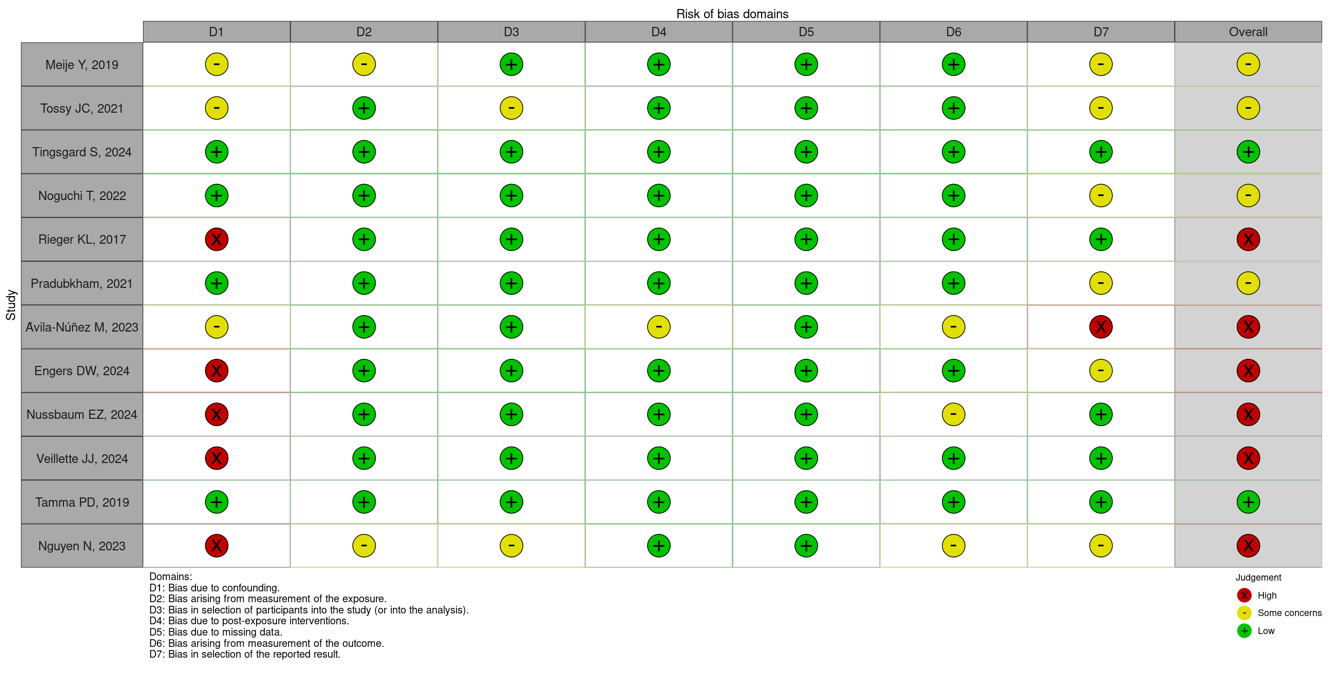
[Randomized trial]**

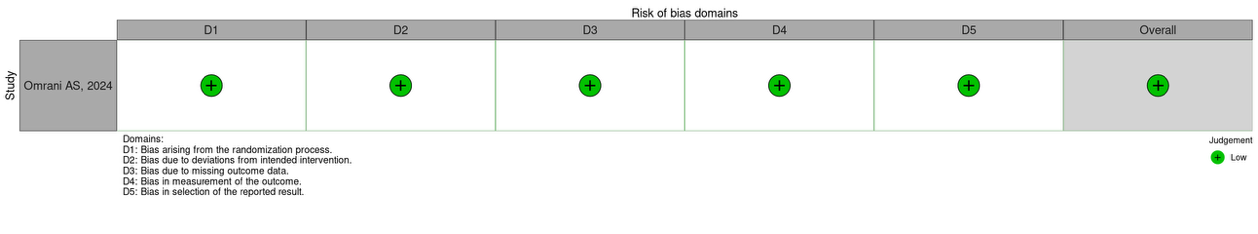


**NOTE.** The risk of bias in three studies (conference abstracts) is not given in the figure (Williams K et al, 2019, Savage H, 2021, and Mulvay N, 2021) because it was unable to be assessed due to insufficient information.

**Supplementary Figure 4.**

Forest plots of the random-effects meta-analysis of the outcomes in the subgroup analysis of studies including immunocompromised patients with a bloodstream infection due to gram-negative bacilli who received either oral stepdown antimicrobial therapy or intravenous antimicrobial therapy

**[Mortality]**

**
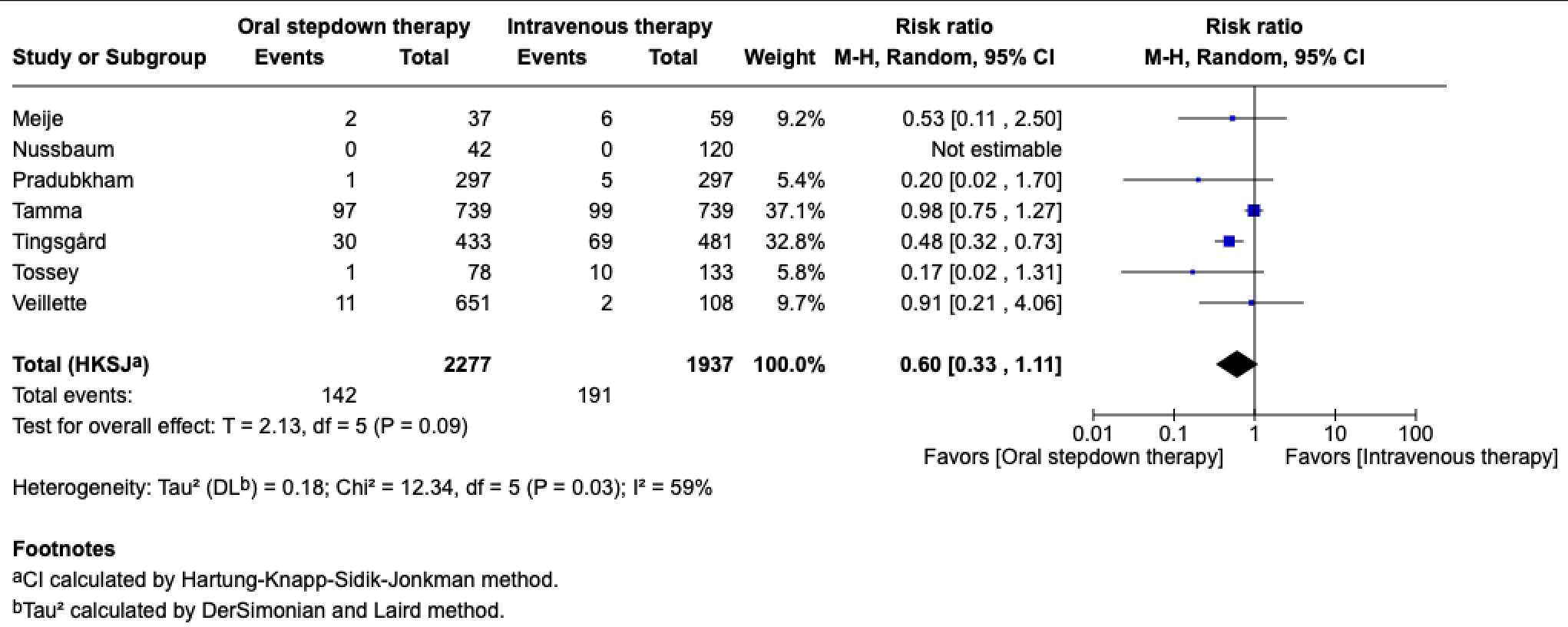
**

**[Treatment failure]**


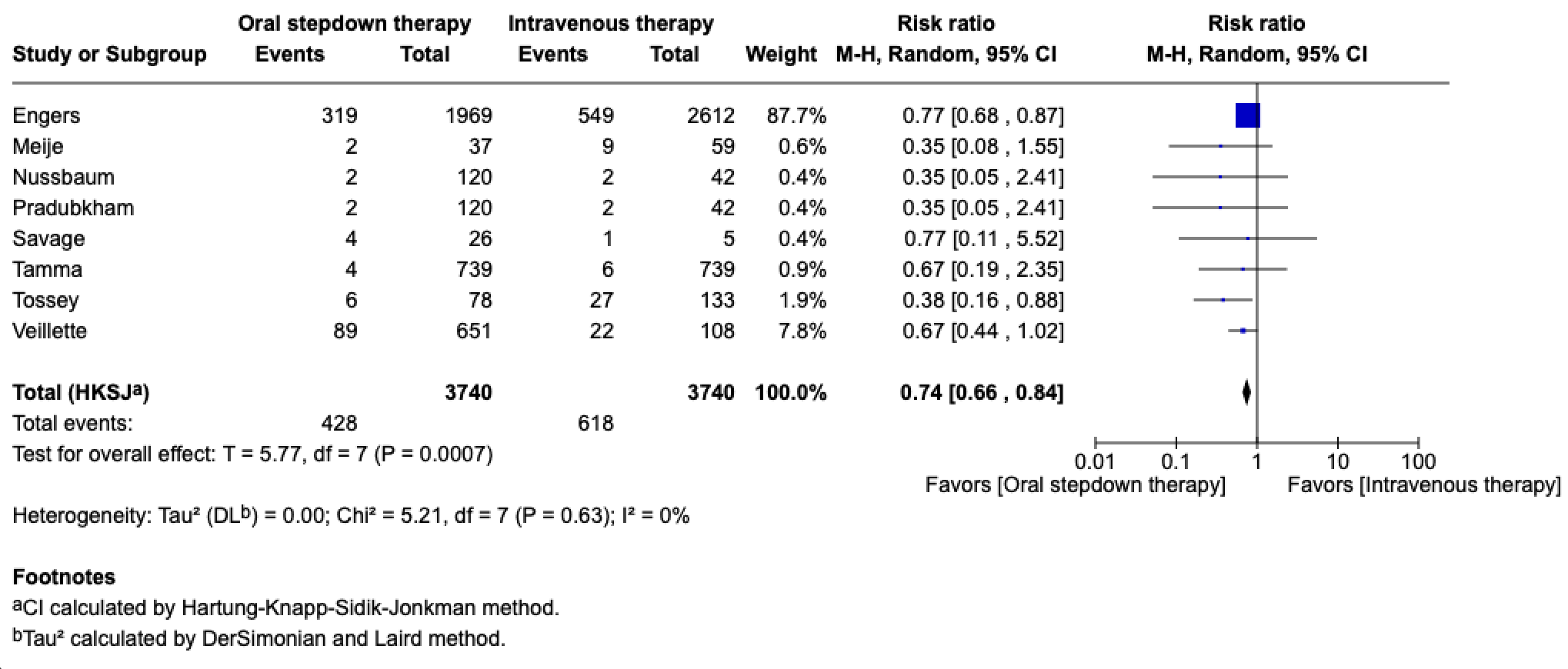


**Supplementary Figure 5.**

Forest plots of the random-effects meta-analysis of the outcomes in patients with a bloodstream infection due to Enterobacterales with multidrug-resistant organisms (Amp C or extended spectrum beta-lactamase producing organisms) who received either oral stepdown antimicrobial therapy or intravenous antimicrobial therapy

**[Mortality]**

**
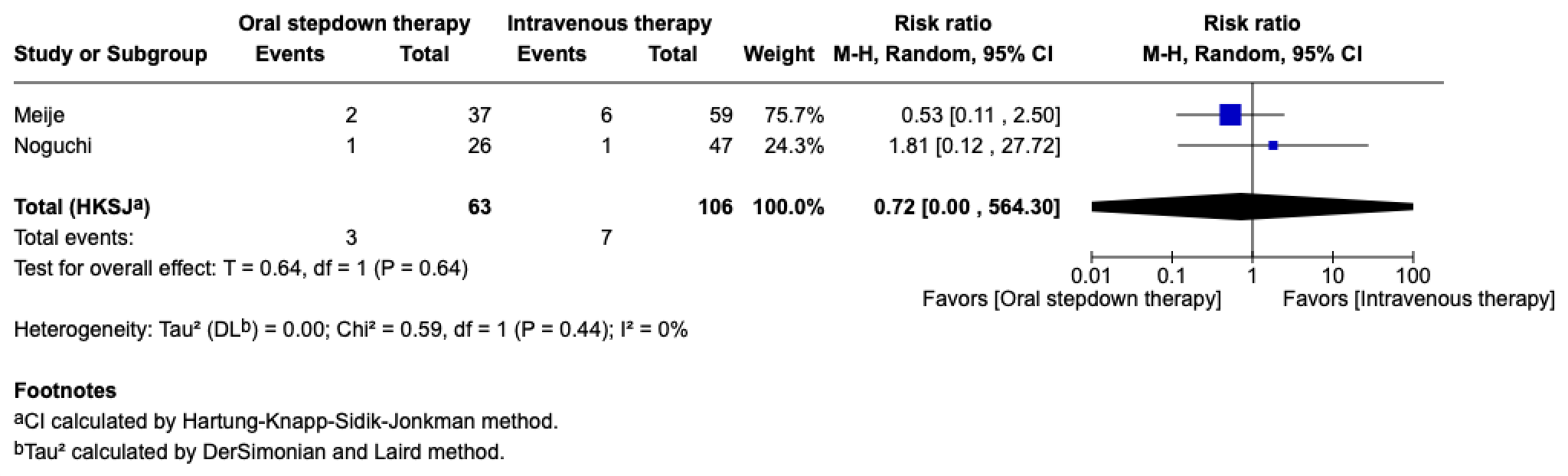
**

**[Treatment failure]**


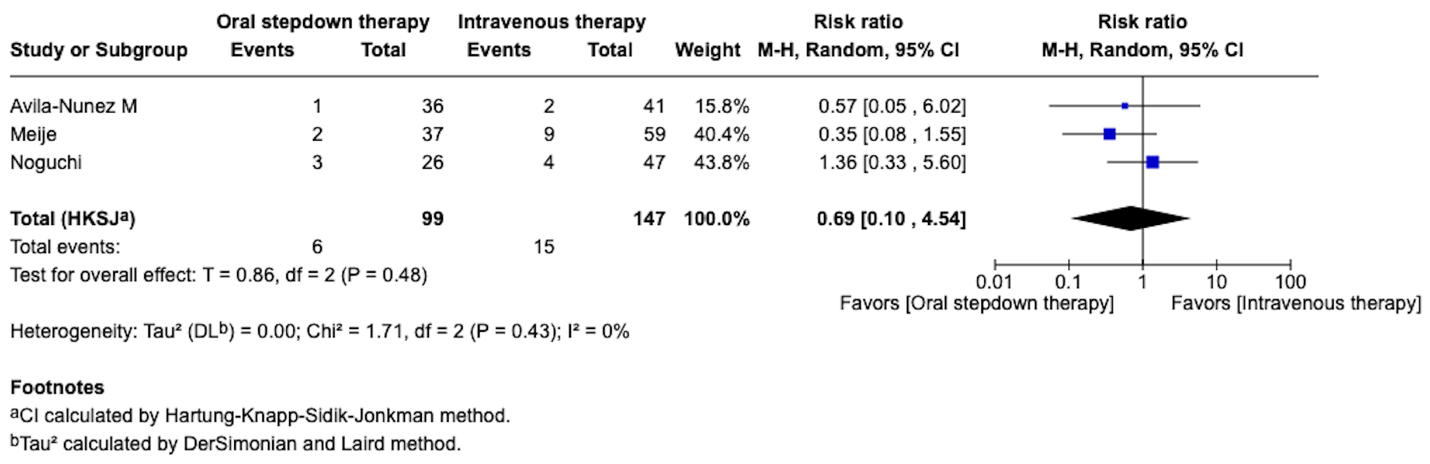


**Supplementary Figure 6.**

Forest plots of the random-effects meta-analysis of the outcomes in patients with a bloodstream infection due to gram-negative bacilli who received either oral stepdown antimicrobial therapy or intravenous antimicrobial therapy, focusing on studies with oral transition within 5 days (median or mean).

**[Mortality]
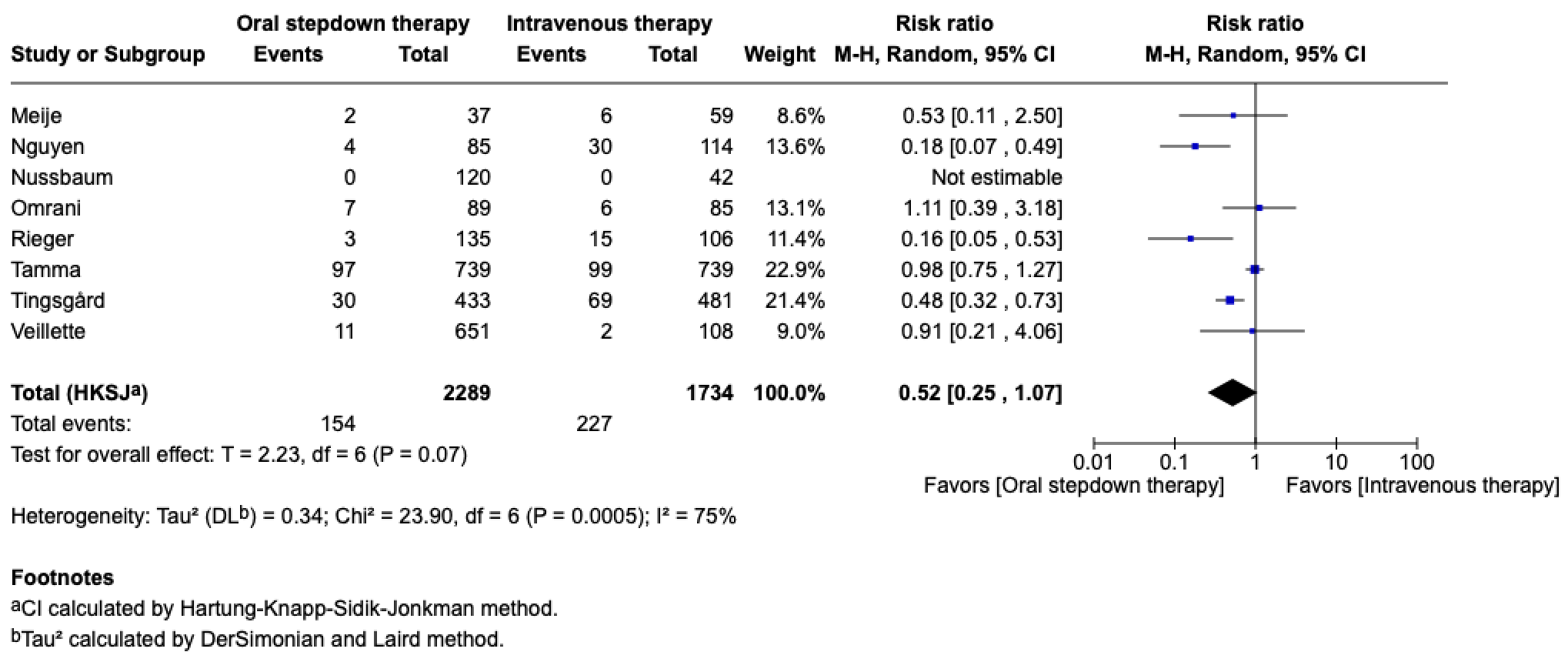
**

**[Treatment failure]**


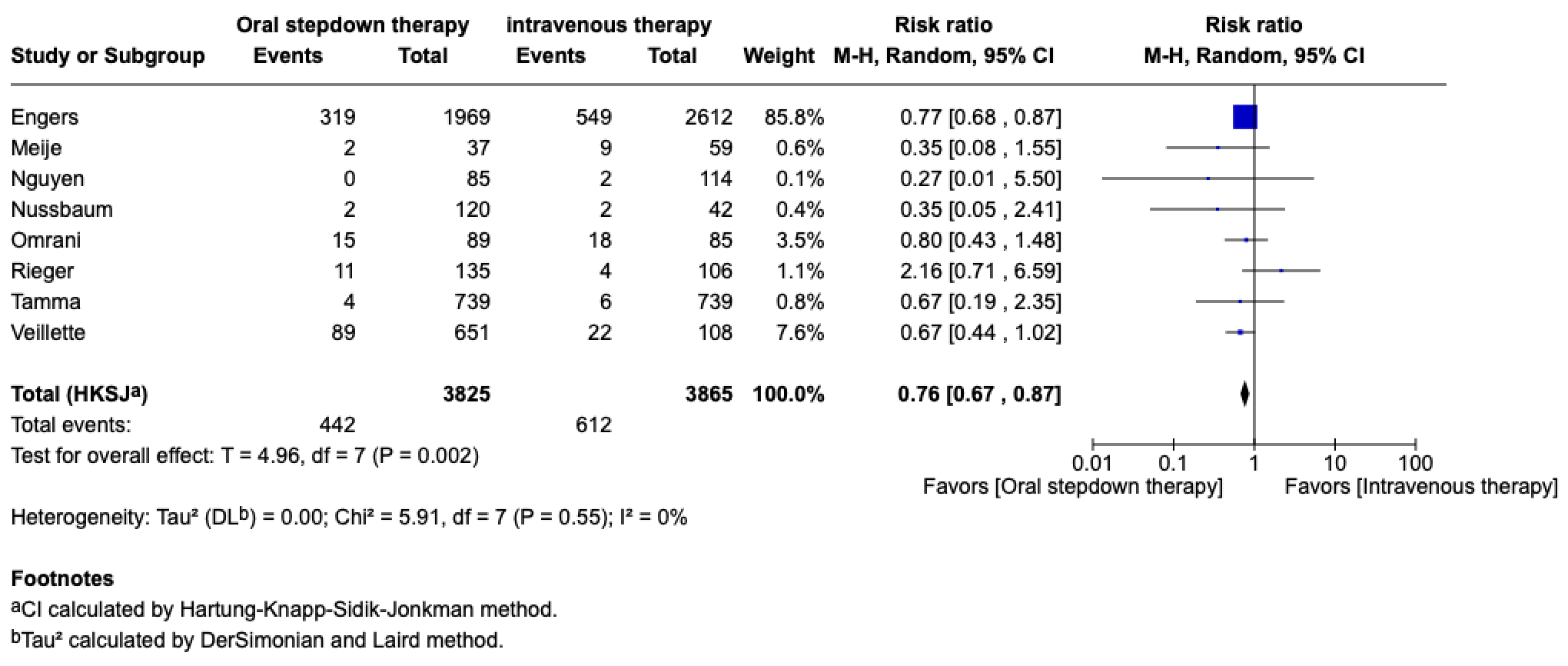


**Supplementary figure 7.**

Forest plots of the random-effects meta-analysis of the outcomes in patients with a bloodstream infection due to gram-negative bacilli who received either oral stepdown antimicrobial therapy or intravenous antimicrobial therapy, focusing on studies with 30-day mortality

**[Mortality]**


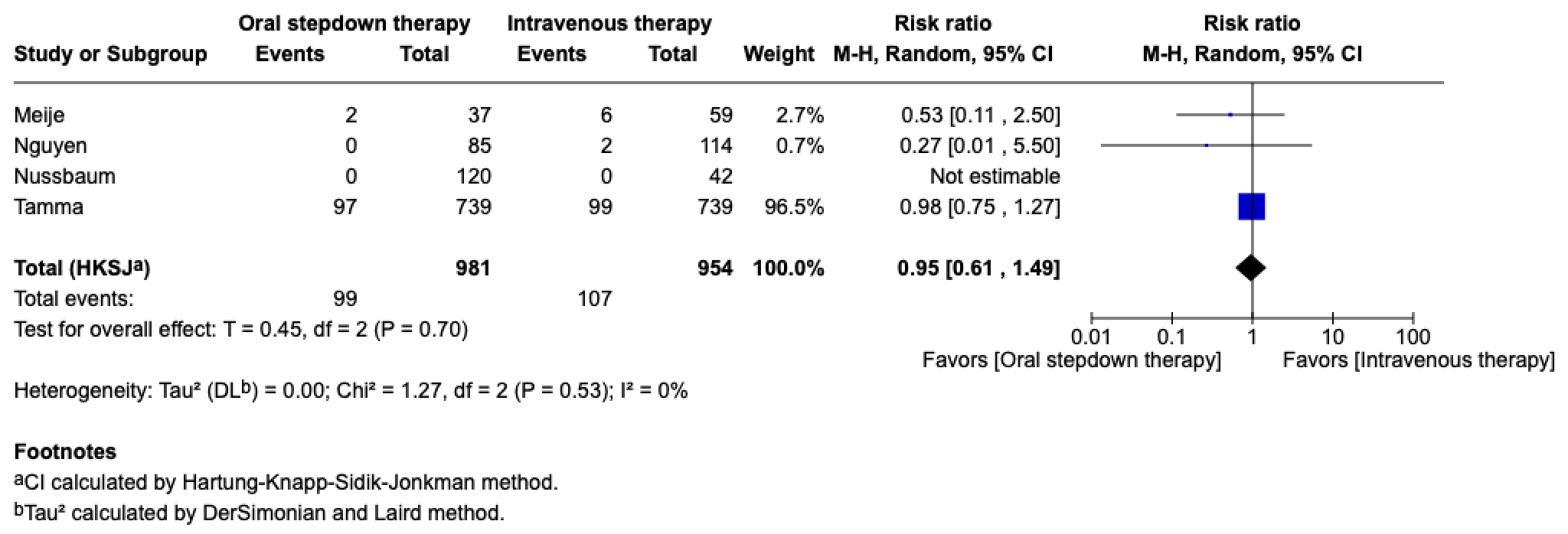


**[Treatment failure]**


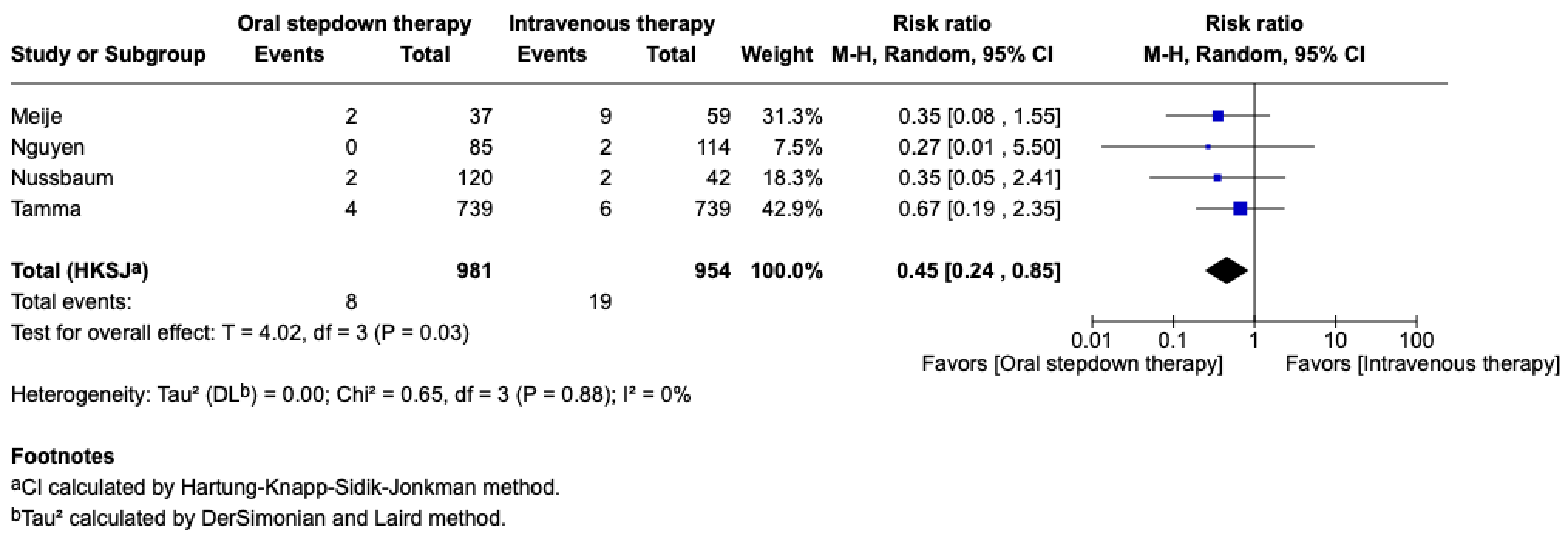


**Supplementary figure 8**

Forest plots of the random-effects meta-analysis of the outcomes in patients with a bloodstream infection due to gram-negative bacilli stemming from a urinary tract infection who received either oral stepdown antimicrobial therapy or intravenous antimicrobial therapy

**[Mortality]
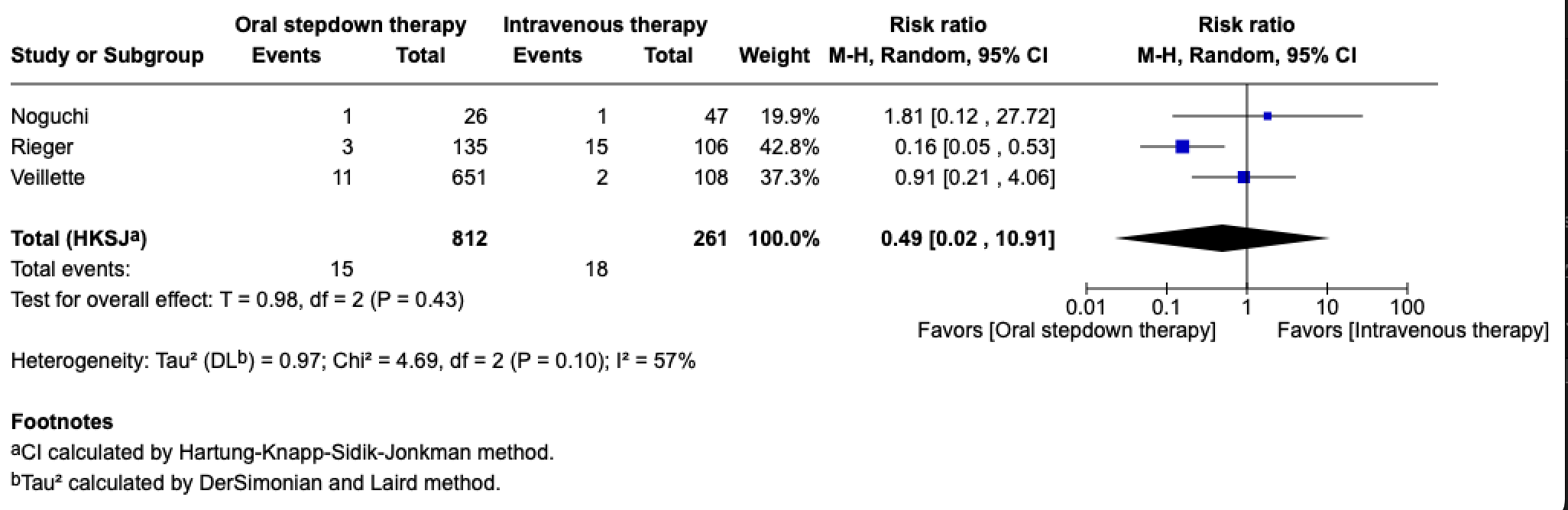
**

**[Treatment failure]**
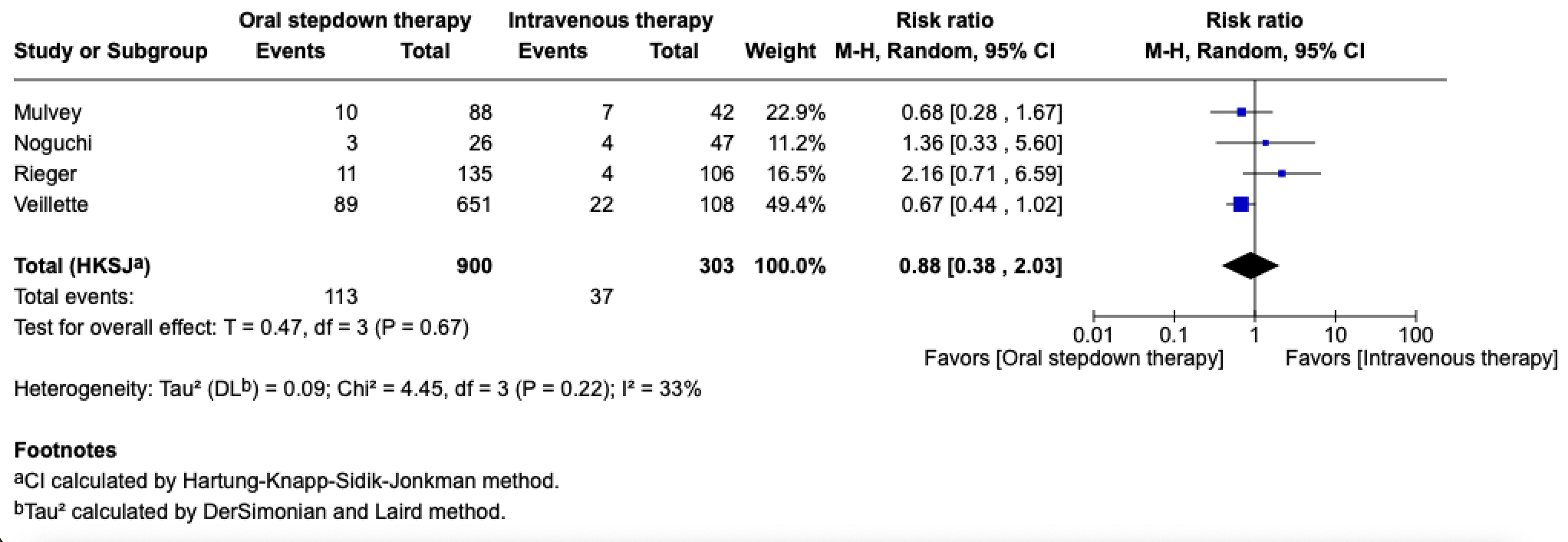

Supplement: Honda et al. supplementary material [file S2732494X26107426sup001.docx]
